# Exploratory analysis of risk factors associated with antimicrobial resistance in urinary *Escherichia coli* from dogs in the United States (2018–2024)

**DOI:** 10.1186/s12917-026-05659-6

**Published:** 2026-06-24

**Authors:** Rasaq A. Ojasanya, J. Scott Weese, Kurtis E. Sobkowich, Anne Deckert, Donald Szlosek, Andy Plum, Theresa M. Bernardo, Zvonimir Poljak

**Affiliations:** 1 Department of Population Medicine, Ontario Veterinary College, University of Guelph, Ontario, Canada; 2 Department of Pathobiology, Ontario Veterinary College, University of Guelph, Ontario, Canada; 3 Centre for Public Health and Zoonoses, Ontario Veterinary College, University of Guelph, Ontario, Canada; 4 IDEXX Laboratories Inc., Westbrook, ME USA

**Keywords:** Antimicrobial resistance, Urinary *Escherichia coli*, Dogs, Risk factors

## Abstract

**Background:**

Bacterial cystitis, predominantly caused by *Escherichia coli*, is a leading cause for antimicrobial prescriptions in dogs. The use of antimicrobials contributes to selection pressure for antimicrobial resistance (AMR), a growing concern for veterinary and public health. This study examined risk factors for resistance in urinary *E. coli* isolates from dogs in the United States, focusing on prior positive cultures and other host, socioeconomic, and geographic factors using data from a national commercial diagnostic laboratory. Associations between selected exposures of interest and AMR were assessed using multilevel logistic regression models, accounting for clustering at the patient, county, and state levels.

**Results:**

A total of 393,972 clinical urinary *E. coli* isolates from 339,977 dogs across the United States, submitted between 2018 and 2024, were tested for antimicrobial susceptibility. Resistance was highest to amoxicillin (27.39%; 95% CI: 27.25–27.53). When examined in multilevel logistic regression models, and across all antimicrobials, the greatest proportion of variance occurred at the patient level (range: 22.2–46.1%). The log odds of detecting resistance increased linearly with the number of prior positive cultures in the previous six months. After adjusting for all other covariates, every 1% increase in the annual county-level percentage of dogs with positive culture and antimicrobial susceptibility test was associated with a 1–2% decrease in the odds of resistance across all antimicrobials (OR range: 0.98–0.99; *p* < 0.001). States with 1,500–1,999 housing units per veterinarian had higher odds of marbofloxacin resistance (OR: 1.85, 95% CI: 1.20–2.85, adjusted *p* = 0.027) than states with 500–999 units, a pattern consistent across other antimicrobials.

**Conclusion:**

Patient-level factors, particularly recent repeated positive cultures examined in this study, are associated with AMR in canine urinary *E. coli*, likely reflecting cumulative antimicrobial exposure and/or chronic resistant infections. Additionally, lower odds of detecting AMR were observed in counties with higher microbiological testing frequency and in states with higher veterinarian density, which may reflect differences in access to veterinary care or diagnostic practices. However, because these variables are measured at the group level, they do not capture individual-level veterinary care. Further research incorporating patient-level measures of care access and prescribing practices will be crucial.

**Supplementary Information:**

The online version contains supplementary material available at 10.1186/s12917-026-05659-6.

## Background

Bacterial cystitis is among the most common bacterial infections encountered in companion animals, particularly in dogs [1]. *Escherichia coli*, consistently identified as the predominant bacterial species, is the most frequently isolated bacterium from cases of canine bacteriuria [2].

Bacterial cystitis is a common reason for prescribing antimicrobials in companion animals, accounting for about 11% of all such prescriptions in dogs [3]. Antimicrobials are essential for treatment, but their use contributes to the risk of antimicrobial resistance (AMR). In most cases, bacterial culture and particularly antimicrobial susceptibility testing (AST) are not performed until recurrent infections occur [4]. AMR can lead to prolonged illnesses, higher treatment costs, and, importantly, the potential for zoonotic transmission of plasmid-mediated resistance genes [1, 5].

AMR surveillance is essential for developing evidence-based strategies that promote antimicrobial stewardship. This is particularly important for underrepresented populations such as companion animals, where available data are often limited, fragmented, or inconsistently reported [6]. Globally, AMR surveillance in companion animals remains relatively sparse, making it challenging to fully understand how resistance emerges, spreads, and persists in this population [7]. Addressing these gaps requires a more holistic approach; one that considers the diverse and interconnected factors driving AMR in companion animals.

Integrating host and ecological factors, and considering how these factors interact, can strengthen causal evidence and contribute to a more comprehensive understanding of the complex drivers of AMR in companion animals. While host-level determinants such as age, sex, prior antimicrobial exposure, and comorbidities are risk factors for resistant infections [8], the relative contribution of these factors compared to broader ecological influences remains unclear. Ecological factors, including geographic location, population density, regional antimicrobial usage patterns, and access to veterinary care, could be equally important in influencing resistance outcomes. A multilevel investigation considering host and ecological factors could offer valuable perspectives on the potential risks and drivers of AMR. Against this backdrop, this present study aims to identify risk factors for AMR in urinary *E. coli* isolates from dogs in the United States by assessing prior positive cultures and other host-related, socioeconomic, and geographic factors, using microbiological data from a national commercial diagnostic laboratory in the United States. The findings of the study aim to generate insights that further strengthen antimicrobial stewardship, support clinical decision-making, and inform One Health–aligned public health strategies.

## Methods

### Data source for urinary *E. coli*

This nationwide retrospective observational study analyzed data from microbiological examinations of clinical urine samples from dogs over seven consecutive years (January 2018 to December 2024). These samples were submitted by veterinary practices across the United States to IDEXX Reference Laboratories, encompassing primary care clinics and specialty care hospitals; however, the data did not differentiate between these levels of care. Urine samples were collected via cystocentesis, catheterization, and the free catch methods. All culture and susceptibility testing procedures were conducted using standardized methods across IDEXX microbiology laboratories nationwide. Isolates were cultured from these samples and subjected to AST using phenotypic methods, as described below. The dataset included the following variables: unique de-identified patient number, date of sample collection (month and year), location of the veterinary practice (county, state, and three-digit ZIP code), patient age, sex, specimen type, organism isolated, and the AST results for all antimicrobials tested.

### Other data sources

Descriptions of the derived or constructed variables and additional data integrated with the IDEXX microbiological dataset are provided in Supplementary Table 1, including a description of each variable, hierarchical level, calculation method (where applicable), and data source. Demographic and socioeconomic data from the United States Department of Agriculture were integrated to provide the most recent county-level human population estimates and county-level median household income for 2022 [9]. The human population estimates were converted to household population figures using the reported average household size of 2.5 individuals per household for 2022 [10].

Pet ownership data were obtained from the American Veterinary Medical Association to estimate county-level dog populations [11]. According to the most recent statistics, 45.5% of U.S. households own dogs, with an average of 1.5 dogs per pet-owning household. These figures were applied to each county’s estimated number of households to derive estimates of the dog population.

The percentage tested represents the annual proportion of dogs with a positive culture (all of which had AST performed) relative to the estimated dog population in each county. This metric reflects county-level variation in testing intensity among clinically diseased animals and may serve as a proxy for diagnostic testing practices. Rural-urban classification was integrated with the microbiological data using the 2023 Rural-Urban Continuum Codes with their corresponding descriptions [12]. Finally, the state-level veterinarian density was incorporated using the number of housing units per veterinarian in each state as of 2018, as described by Ouedraogo et al. [13].

### Bacterial identification and antimicrobial susceptibility testing

*E. coli* was isolated from urine cultures, and the isolates were identified using Matrix-Assisted Laser Desorption/Ionization Time-Of-Flight mass spectrometry (MALDI-TOF Biotyper; Bruker, Billerica, MA, USA). AST was performed on the VITEK system (bioMérieux, Inc., Durham, NC), using the GN-98 card [14]. In instances where the VITEK system was not available, AST was performed using the Kirby-Bauer disk diffusion method. The bacterial inoculum was suspended in sterile saline and adjusted to a 0.5 McFarland turbidity standard before inoculation onto Mueller–Hinton agar plates. Antimicrobial disks were applied within 15 min of inoculation, and plates were incubated at 35 °C for 16–18 h before zone diameters were measured [15]. *E. coli* isolates were included in the study only if they had complete susceptible, intermediate, or resistant results for the complete panel of antimicrobials tested concurrently: amoxicillin (inferred from ampicillin testing, used as a surrogate), amoxicillin-clavulanic acid, trimethoprim-sulfamethoxazole, marbofloxacin, enrofloxacin, cefpodoxime, and cefovecin, which reflect commonly used drugs for treatment of bacterial cystitis [1], as illustrated in the flowchart provided in Supplementary Fig. 1. AST results were reported as “susceptible,” “resistant,” or “intermediate,” by the diagnostic laboratory, based on the bacterial and animal species-specific minimum inhibitory concentration breakpoints established by the Clinical and Laboratory Standards Institute [16]; the specific breakpoints applied are provided in Supplementary Table 2.

### Data management

The data were validated for consistency, and records from the same animal patient, date of sample collection, location of the veterinary practice, patient age, patient sex, specimen type, organism isolated, and the AST results for all antimicrobials tested were deemed duplicate tests and were excluded from the analysis. Other urinary specimen types, such as bladder walls and uroliths (bladder stones), were excluded from the analysis because of their small sample sizes and the possibility that they have different breakpoints, which are not comparable with those of standard urinary specimens. The patients’ ages were categorized into four groups: ≤1 year (puppy), 2–6 years (adult), 7–11 years (senior), and ≥ 12 years (geriatric) [17].

### Isolates inclusion and exclusion criteria

The number of prior positive cultures within the preceding six months (NPPC6) was calculated for each patient based on their unique identifier and sample submission history (year and month). The first observation for each patient was assigned a value of 0, as no prior tests existed, while subsequent observations were assigned positive values if one or more prior tests occurred within the preceding six months. All available observations were used to derive this event-level exposure variable. To ensure comparability across observations, those occurring within the first six months of the study period were excluded from the analytic dataset due to insufficient prior history. This restriction reduced potential misclassification and mitigated bias arising from left censoring, which could otherwise lead to underestimation of prior culture exposure.

### Study variables and hierarchical structure

Study variables were hierarchically structured across four levels. At the event level, variables encompassed age, month, year, and NPPC6. The subsequent animal level incorporated patient sex. At the county level, variables captured contextual and socioeconomic characteristics, including the Rural-Urban Continuum Codes, median household income, and the percentage tested. Included at the state level was the number of housing units per veterinarian (a proxy for veterinarian density).

### Causal framework and variable selection

To support causal inference and identify the minimum set of variables required for adjustment, a directed acyclic graph (DAG) was constructed (*DAGitty* package in R), representing the hypothesized relationships between the exposures of interest and AMR [18]. This DAG was informed by existing literature, when possible, enabling a systematic assessment of potential confounders and guiding the selection of covariates necessary to estimate the effect of selected exposures on AMR. In accordance with the methodological recommendations of Westreich and Greenland [19], adjusted effect estimates are presented after controlling for potential confounding factors.

The primary exposure of interest was NPPC6. The minimal sufficient adjustment set derived from the DAG for this exposure included month, year, age, median household income, Rural-Urban Continuum Codes, sex, the percentage tested, and veterinarian density. We fitted three sets of models. Model set 1 evaluated the total effect of NPPC6 on AMR, adjusting for year, month, age, sex, age–sex interaction, Rural-Urban Continuum Codes, median household income, and veterinarian density, but excluding the percentage tested. Model set 2 assessed the effect of age and sex without adjusting for any covariate to estimate the total effect of these variables on AMR. Model set 3 examined the direct effects of all covariates (including year, month, age, sex, age–sex interaction, Rural-Urban Continuum Codes, veterinarian density, percentage tested, NPPC6, and median household income) on AMR.

### Statistical analysis

Statistical analyses were conducted using R software [20]. For each antimicrobial, 95% confidence intervals for outcome proportions were estimated using the Wald method.

The AST outcome for each antimicrobial was dichotomized by grouping isolates classified as intermediate or resistant (*R* = 1), and comparing them with those classified as susceptible (*R* = 0), consistent with most epidemiological studies on AMR [7]. This binary outcome was first modeled using a null (intercept-only) model, followed by univariable and multivariable mixed-effect logistic regression models. All models included random intercepts at the patient, county, and state levels to account for hierarchical clustering within the data structure.

Univariable analyses were conducted to assess the strength, direction, and significance of each of the variables on AMR. These sets were further evaluated within a multivariable mixed-effects logistic regression framework, with statistical significance determined using partial likelihood ratio tests by comparing full models (including the variable of interest) to reduced models (excluding it). Model fit was compared using the Akaike Information Criterion and Bayesian Information Criterion. All multivariable model sets incorporated an interaction term between age and sex. Unknown categories within the age and sex variables were retained in all models to preserve observations with missing demographic information and minimize data loss. However, only estimates for the biologically interpretable sex categories (male and female) and known age groups are presented. Model fit was evaluated by comparing observed and grouped marginal predicted probabilities obtained from the mixed-effects logistic regression model, and the global goodness-of-fit of the resulting population-average was assessed using the le Cessie–van Houwelingen–Copas–Hosmer unweighted sum-of-squares test, and statistical significance was defined as *p* < 0.05 [21]. We additionally and separately applied a penalized likelihood ratio approach with Firth correction to assess the robustness of estimates. Post hoc pairwise comparisons were performed using the Tukey method, which provides greater statistical power and is less conservative than other multiple comparison procedures [22].

## Results

### Study population, isolates characteristics, and antimicrobial susceptibility profiles

Overall, 421,354 urinary *E. coli* isolates were recovered from 365,641 individual canine patients. Of these, 27,382 isolates (from 26,289 dogs) were excluded because they ocurred within the first six months of the study period. This left 393,972 isolates (from 339,977 dogs) for inclusion in the analysis. Most were from female dogs (77.92%, *n* = 306,972). Isolates recovered from senior (36.11%, *n* = 142,253) and geriatric (36.86%, *n* = 145,213) dogs made up the largest age group. Among all isolates, 10.23% were from canine patients with at least one prior positive urinary culture within the past six months (NPPC6 ≥ 1); most had only one prior positive culture (79.94%, *n* = 32,214), and the rest had two or more. Most isolates were recovered from dogs in Rural-Urban Continuum Code 1 counties (metro counties with 1 million people or more) (66.96%, *n* = 263,799), upper-middle-income areas ($70k to less than $100k) (53.40%, *n* = 210,385), and states with 1,000 and 1,499 housing units per veterinarian (65.87%, *n* = 259,521). The frequency distribution of the isolate characteristics is presented in Table 1.

**Table 1:** Descriptive characteristics of urinary *E. coli* isolates from dogs in the USA, including patient, socioeconomic, and geographic variables

Sex	*n* (%)
Female	306,972 (77.92)
Male	85,409 (21.68)
Unknown	1,591 (0.40)
Age group	
1 year or less (Puppy)	41,594 (10.56)
2 years to 6 years (Adult)	63,738(16.18)
7 years to 11 years (Senior)	142,253 (36.11)
12 years and above (Geriatric)	145,213 (36.86)
Unknown	1,174 (0.30)
Isolates from patients with at least one prior positive culture from the last 6 months	
No (0)	353,675 (89.77)
Yes (1)	40,297 (10.23)
Number of prior positive cultures from the last 6 months	
1	32,214 (79.94)
2	6,273 (15.57)
3	1,404 (3.48)
4	340 (0.84)
5	66 (0.17)
Rural-Urban Continuum Codes	
1-Metro-counties with 1 million people or more	263,799 (66.96)
2-Metro-counties with 250k to 1 million people	79,462 (20.17)
3-Metro-counties with fewer than 250k people	26,455 (6.71)
4-Non-metro counties with 20k or more people, adjacent to a metro area	10,735 (2.72)
5-Non-metro counties with 20k or more, not adjacent to a metro area	2,290 (0.58)
6-Non-metro counties with 5k to 20k people, adjacent to a metro area	5,809 (1.47)
7-Non-metro counties with 5k to 20k people not adjacent to metro area	2,764 (0.70)
8-Non-metro counties with fewer than 5k people, adjacent to a metro area	1,802 (0.46)
9-Non-metro counties with fewer than 5k people, not adjacent to a metro area	856 (0.22)
Median household income	
Low (Less than $40k)	121 (0.03)
Lower-Middle ($40k to less than $70k)	90,914 (23.08)
Upper-Middle ($70k to less than $100k)	210,385 (53.40)
High (Greater than or equal to $100k)	92,552 (23.49)
Number of housing units per veterinarian at the state level	
500–999	40,045 (10.16)
1,000–1,499	259,521 (65.87)
1,500–1,999	92,511 (23.48)
2,000–3,000	1,895 (0.48)

Of the 393,972 urinary *E. coli* isolates analyzed, resistance was highest for amoxicillin (27.39%, *n* = 107,898; 95% CI: 27.25–27.53) and lowest for trimethoprim-sulfamethoxazole (7.13%, *n* = 28,087; 95% CI: 7.05–7.21). Descriptive statistics of the AST profiles are provided in Supplementary Table 3.

### Proportion of variance and univariable analyses

Variance partitioning from the null multilevel models revealed that most of the variability in AMR of urinary *E. coli* from dogs occurred at the patient level, ranging from 22.2% to 46.1% of the total variance across antimicrobials, followed by the state level and, finally, the county level (Table 2).

**Table 2:** Variance components and proportion of total variance explained by random effects in the null mixed effect models for the antimicrobial resistance of urinary *E. coli* isolates from dogs

		Variance estimate (Proportion of total variance explained by random effect (%))						
Random Effect	Level	AMOXI	AMOCLA	TRISUL	CEFOV	CEFPOD	ENROF	MARBOF
Intercept	State (*n* = 51)	0.04 (0.9)	0.07 (1.5)	0.16 (2.8)	0.09 (1.9)	0.10 (2.1)	0.24 (3.9)	0.37 (5.1)
Intercept	County (*n* = 1,823)	0.03 (0.7)	0.06 (1.3)	0.08 (1.4)	0.06 (1.3)	0.07 (1.5)	0.16 (2.6)	0.23 (3.2)
Intercept	Patient (*n* = 393,972)	0.96 (22.2)	1.10 (24.3)	2.20 (38.4)	1.18 (25.5)	1.24 (26.4)	2.45 (39.9)	3.33 (46.1)
	Residual variance	3.29	3.29	3.29	3.29	3.29	3.29	3.29
	Total variance	4.32	4.52	5.73	4.62	4.70	6.14	7.22
State level includes the District of Columbia								

*AMOXI *Amoxicillin, *AMOCLA *Amoxicillin clavulanic acid, *TRISUL *Trimethoprim-sulfamethoxazole, *CEFOV *Cefovecin, *CEFPOD *Cefpodoxime, *ENROF *Enrofloxacin, *MARBOF *Marbofloxacin

In the univariable analysis, most variables were significantly associated with AMR (*p* < 0.05). However, sex was not significantly associated with resistance to amoxicillin (*p* = 0.412), Rural–Urban Continuum Codes were not associated with marbofloxacin (*p* = 0.296), and median household income was not associated with amoxicillin (*p* = 0.653) or trimethoprim–sulfamethoxazole (*p* = 1.000). Detailed univariable results are provided in Supplementary Table 4.

### Total effect of exposures of interest on antimicrobial resistance

Model sets 1, 2, and 3 demonstrated good fit and calibration, with no significant differences between the grouped predicted probabilities and observed outcomes. Penalized likelihood estimates were consistent in magnitude and direction with the estimates from these models, supporting the robustness of the results.

From Model set 1, there was a strong positive linear association between NPPC6 and the log odds of detecting AMR. As NPPC6 for urinary *E. coli* increased, the log odds of resistance rose across all antimicrobials compared with isolates from patients without NPPC6. The strongest associations were observed for fluoroquinolones: isolates from patients with five prior positive cultures had 8.28 times higher odds of resistance to enrofloxacin (95% CI: 3.95–17.35) and 12.37 times higher odds of resistance to marbofloxacin (95% CI: 5.79–26.44) compared with isolates from patients without NPPC6. The multivariable model estimating the total effect of NPPC6 on AMR is presented in Table 3, with pairwise contrasts revealing similar patterns (Supplementary Table 5).

**Table 3:** Multivariable analysis of the total effect of the number of prior positive cultures from the last six months on the antimicrobial resistance of urinary *E. coli* isolates from dogs in the USA

Antimicrobial	Number of prior positive cultures from the last 6 months	Estimates	Standard Error	OR (95% CI)	*p*-value	Partial χ²	df	χ²/df	Partial likelihood ratio test
	0	Ref.							
	1	0.252	0.014	**1.29 (1.25–1.32)**	**< 0.001**				
AMOXI	2	0.537	0.030	**1.71 (1.61–1.82)**	**< 0.001**	983.23	5	196.65	**< 0.001**
	3	0.859	0.065	**2.36 (2.08–2.68)**	**< 0.001**				
	4	1.076	0.140	**2.93 (2.22–3.87)**	**< 0.001**				
	5	1.847	0.369	**6.34 (3.08–13.03)**	**< 0.001**				
	0	Ref.							
	1	0.241	0.016	**1.27 (1.23–1.31)**	**< 0.001**				
	2	0.530	0.033	**1.70 (1.59–1.82)**	**< 0.001**	789.68	5	157.94	**< 0.001**
AMOCLA	3	0.812	0.067	**2.25 (1.97–2.56)**	**< 0.001**				
	4	0.989	0.139	**2.69 (2.05–3.53)**	**< 0.001**				
	5	1.023	0.314	**2.78 (1.51–5.10)**	** 0.001**				
	0	Ref.							
	1	0.537	0.022	**1.71 (1.64–1.78)**	**< 0.001**				
TRISUL	2	0.926	0.042	**2.52 (2.32–2.74)**	**< 0.001**	1546.60	5	309.32	**< 0.001**
	3	1.162	0.083	**3.20 (2.72–3.77)**	**< 0.001**				
	4	1.386	0.164	**4.00 (2.90–5.52)**	**< 0.001**				
	5	1.229	0.370	**3.42 (1.66–7.08)**	**< 0.001**				
	0	Ref.							
	1	0.287	0.016	**1.33 (1.29–1.38)**	**< 0.001**				
	2	0.626	0.034	**1.87 (1.75–2.00)**	**< 0.001**				**< 0.001**
CEFOV	3	0.956	0.068	**2.60 (2.27–2.97)**	**< 0.001**	1014.95	5	202.99	
	4	1.023	0.140	**2.78 (2.14–3.61)**	**< 0.001**				
	5	1.087	0.321	**2.97 (1.58–5.58)**	**< 0.001**				
	0	Ref.							
	1	0.241	0.017	**1.27 (1.23–1.32)**	**< 0.001**				
CEFPOD	2	0.552	0.035	**1.74 (1.62–1.87)**	**< 0.001**	747.48	5	149.50	**< 0.001**
	3	0.862	0.070	**2.37 (2.06–2.72)**	**< 0.001**				
	4	0.851	0.144	**2.34 (1.77–3.09)**	**< 0.001**				
	5	0.958	0.327	**2.61 (1.37–4.94)**	** 0.003**				
	0	Ref.							
	1	0.724	0.019	**2.06 (1.98–2.13)**	**< 0.001**				
ENROF	2	1.245	0.037	**3.47 (3.22–3.75)**	**< 0.001**	3616.46	5	723.29	**< 0.001**
	3	1.623	0.077	**5.07 (4.36–5.90)**	**< 0.001**				
	4	1.982	0.160	**7.26 (5.31–9.92)**	**< 0.001**				
	5	2.113	0.377	**8.28 (3.95–17.35)**	**< 0.001**				
	0	Ref.							
	1	0.760	0.021	**2.14 (2.05–2.23)**	**< 0.001**				
MARBOF	2	1.277	0.041	**3.58 (3.30–3.89)**	**< 0.001**	3250.67	5	650.13	**< 0.001**
	3	1.722	0.082	**5.60 (4.77–6.57)**	**< 0.001**				
	4	1.888	0.167	**6.61 (4.77–9.15)**	**< 0.001**				
	5	2.516	0.388	**12.37 (5.79 − 26.44)**	**< 0.001**				

Model adjusted for month, year, age, sex, age–sex interaction, Rural-Urban Continuum Codes, median household income, and veterinarian density, with random effects at the state, county, and patient levels

Inverse OR values (1/OR) aid interpretation when the reference category is reversed

Ref. – Isolates from patients without prior positive culture from the last 6 months

χ² - Chi-square statistic

df - degrees of freedom

*AMOXI* Amoxicillin, *AMOCLA* Amoxicillin clavulanic acid, *TRISUL* Trimethoprim-sulfamethoxazole, *CEFOV* Cefovecin, *CEFPOD* Cefpodoxime, *ENROF* Enrofloxacin, *MARBOF* Marbofloxacin

Bolded ORs, 95% CIs, *p*-values, and partial likelihood ratio test *p*-values indicate statistical significance at *p* < 0.05

From Model set 2, pairwise comparisons of the age–sex interaction indicated that among senior and geriatric dogs, isolates from female patients generally had higher odds of resistance than isolates from males, except for trimethoprim–sulfamethoxazole. Among adult male dogs, there are higher odds of resistance to trimethoprim–sulfamethoxazole (OR 1.17; 95% CI: 1.08–1.30; adjusted *p* = 0.003) and marbofloxacin (OR 1.20; 95% CI: 1.10–1.33; adjusted *p* = 0.006) compared with females. Finally, among puppies, isolates from males had higher odds of AMR than those from females (OR range: 1.35–2.27; adjusted *p* < 0.001) (Supplementary Table 6).

### Direct effect of associated risk factors on antimicrobial resistance

From Model set 3, the full multivariable model estimating the direct effects of all covariates on AMR is presented in Supplementary Table 7. Subsets of these variables are shown in separate tables due to the large size of the full model and the pairwise comparisons conducted.

After adjusting for all other covariates, Rural-Urban Continuum Codes had no association with AMR in urinary *E. coli* isolates, except for amoxicillin–clavulanic acid and trimethoprim–sulfamethoxazole. Compared with Rural-Urban Continuum Codes 1 (Metro-counties with 1 million people or more), isolates from dogs in Rural-Urban Continuum Codes 3 (Metro-counties with fewer than 250k people) (OR: 0.92; 95% CI: 0.86–0.99) and Rural-Urban Continuum Codes 9 (Non-metro counties with fewer than 5k people, not adjacent to a metro area) (OR: 0.78; 95% CI: 0.61–0.99) were associated with small decrease in the odds of resistance to amoxicillin–clavulanic acid. In contrast, isolates from dogs in Rural-Urban Continuum Codes 6 (Non-metro counties with 5k to 20k people, adjacent to a metro area) (OR: 1.16; 95% CI: 1.01–1.33) and Rural-Urban Continuum Codes 7 (Non-metro counties with 5k to 20k people not adjacent to metro area) (OR: 1.41; 95% CI: 1.17–1.70) were associated with higher odds of resistance to trimethoprim–sulfamethoxazole (Supplementary Table 8).

After adjusting for all other covariates, median household income was associated with AMR in urinary *E. coli* isolates, but only for select antimicrobials. Compared with high-income counties (greater than or equal to $100k), isolates from dogs in lower-middle income counties ($40k to less than $70k) were associated with small decreases in the odds of resistance to amoxicillin (OR: 0.93; 95% CI: 0.88–0.99), amoxicillin–clavulanic acid (OR: 0.92; 95% CI: 0.85–0.99), and cefpodoxime (OR: 0.90; 95% CI: 0.82–0.98). (Supplementary Table 9).

After adjusting for all other covariates, every 1% increase in the annual county-level percentage of dogs with positive culture and AST, relative to the estimated dog population, was associated with a 1–2% decrease in the odds of resistance across all antimicrobials (OR range: 0.98–0.99; *p* < 0.001) (Table 4).

**Table 4:** Direct effect of annual percentage of dogs with positive cultures and antimicrobial susceptibility testing at the county-level relative to estimated dog population on antimicrobial resistance in urinary *E. coli* isolates from dogs in the USA

Antimicrobial	Estimates	Standard Error	OR (95% CI)	*p*-value	Partial χ²	df	χ²/df	Partial likelihood ratio test
AMOXI	-0.010	0.001	**0.99 (0.99–0.99)**	**< 0.001**	93.13	1	93.13	< 0.001
AMOCLA	-0.010	0.001	**0.99 (0.98–0.99)**	**< 0.001**	65.86	1	65.86	**< 0.001**
TRISUL	-0.015	0.002	**0.99 (0.98–0.99)**	**< 0.001**	63.70	1	63.70	**< 0.001**
CEFOV	-0.010	0.001	**0.99 (0.99–0.99)**	**< 0.001**	56.44	1	56.44	**< 0.001**
CEFPOD	-0.010	0.002	**0.99 (0.99–0.99)**	**< 0.001**	41.01	1	41.01	**< 0.001**
ENROF	-0.016	0.002	**0.98 (0.98–0.99)**	**< 0.001**	78.55	1	78.55	**< 0.001**
MARBOF	-0.020	0.002	**0.98 (0.98–0.99)**	**< 0.001**	83.79	1	83.79	**< 0.001**

Model adjusted for year, month, age, sex, age and sex interaction, Rural-Urban Continuum Codes, median household income, veterinarian density, and number of prior positive cultures from the last 6 months with random effects at the state, county, and patient levels

χ² - Chi-square statistic

df - degrees of freedom

*AMOXI *Amoxicillin, *AMOCLA *Amoxicillin clavulanic acid, *TRISUL *Trimethoprim-sulfamethoxazole, *CEFOV *Cefovecin, *CEFPOD *Cefpodoxime, *ENROF *Enrofloxacin, *MARBO *Marbofloxacin

After adjusting for all other covariates, states with 1,500–1,999 housing units per veterinarian had higher odds of resistance to all antimicrobials compared with states with 500–999 units, with the strongest associations observed for fluoroquinolones: enrofloxacin (OR: 1.69, 95% CI: 1.19–2.40, adjusted *p* = 0.019) and marbofloxacin (OR: 1.85, 95% CI: 1.20–2.85, adjusted *p* = 0.027) (Supplementary Table 10).

## Discussion

In this study, urinary *E. coli* isolates from dogs in the USA exhibited varying levels of resistance to all tested antimicrobials, with high resistance to amoxicillin, a commonly recommended first-line treatment for bacterial cystitis [1]. Previous studies reported higher resistance rates in California between 2010 and 2013, where only 59% of isolates were susceptible to amoxicillin (inferred from ampicillin testing, used as a surrogate; ~41% resistant) [23], followed by even lower susceptibility in the Midwestern United States between 2013 and 2017, where 53% of isolates were susceptible (inferred from ampicillin testing; ~47% resistant) [24]. Differences between these findings and the present study may reflect variation in source populations [25] or differences in the application of antimicrobial susceptibility breakpoints. Additionally, these differences may partly be explained by temporal changes, including increased awareness and adoption of antimicrobial use guidelines for companion animals among veterinarians, which significantly rose from 12% in 2015 to 60% in 2022 across North America [26, 27].

The largest source of variation in AMR was observed between patients as compared to county- or state-level factors, suggesting that the patient level is the most suitable target for antimicrobial stewardship intervention efforts. This finding could reflect biological or sampling factors. From a biological standpoint, we had a limited number of covariates to explain this variability, with the main exposure of interest being NPPC6. This exposure demonstrated a strong positive linear association: a higher number of NPPC6 was consistently associated with increased log odds of resistance across all antimicrobials. A higher number of positive cultures within the past six months could be indicative of chronic or recurrent infections requiring antimicrobial therapy; this alone could drive the observed increase in the odds of detecting AMR. However, other explanations are also possible. For instance, infection with a resistant *E. coli* strain, which requires ongoing management involving diagnostic testing (captured in our dataset), prior antimicrobial therapy, and clinic-level diagnostic practices such as follow-up cultures, rather than representing unique infection episodes (for which data were unavailable). This important question could not be fully explored with the current dataset, which includes only positive diagnostic test results. Future work linking temporal data with medical records and prescription histories would be essential to establish a complete chronology of events. While the large proportion of variance at the patient level is biologically plausible, this finding should be interpreted with caution. From a sampling standpoint, canine patients with severe or hard-to-treat infections caused by resistant bacteria may be tested multiple times. In contrast, those with mild infections may be tested only once or not at all. This differential testing could artificially inflate the proportion of variance at the patient level beyond what we can expect in the source population. One potential solution is to consider clinical episodes as the unit of analysis, rather than individual tests, and select only the first isolate from each episode. Again, linking laboratory results with clinical and prescription data would enhance the feasibility and the validity of this approach. Overall, these findings underscore the importance of point-of-care interventions grounded in antimicrobial stewardship principles, including preventive measures to reduce infection risk, evidence-based prescribing guided by culture and susceptibility testing, and continuous monitoring of treatment outcomes [28].

The increased odds of detecting AMR among senior and geriatric female dogs may be partly explained by their higher lifetime risk of bacterial cystitis compared with males [8]. This risk increases with advancement in age, as older pets are more likely to develop bacterial cystitis [29], potentially resulting in repeated antimicrobial exposure and, consequently, a higher likelihood of AMR detection. However, this pattern is not only specific to cystitis; any disease that has previously required antibiotic treatment could contribute to the cumulative antimicrobial exposure. In male dogs, bacterial cystitis is less common but often more complicated, requiring longer or repeated antimicrobial treatments, which can also select for resistant *E. coli* [8]. Interestingly, in this study, urinary *E. coli* isolates from male puppies exhibited unexpectedly higher odds of resistance across all antimicrobials. The underlying reasons for this pattern remain unclear, as prior clinical and treatment histories were unavailable in our dataset. It is therefore not possible to determine whether these male puppies were more likely to represent complicated infections, previously treated cases, or recent surgical procedures such as neutering, which could partly explain the observed association.

As compared to Rural–Urban Continuum Codes 1, isolates from dogs in Rural–Urban Continuum Codes 3 and 9 had lower odds of resistance to amoxicillin–clavulanic acid, which may reflect reduced selection pressure due to less frequent use of this broader-spectrum antimicrobial agent, fewer cumulative antimicrobial exposures, or more conservative prescribing practices in small-metro and remote non-metro areas. In contrast, Rural–Urban Continuum Codes 6 and 7 showed increased odds of detecting resistance to trimethoprim–sulfamethoxazole, and together, this may reflect differences in prescribing practices, diagnostic access, and antimicrobial availability in smaller or remote communities, where empirical use of trimethoprim–sulfamethoxazole may be more common. The elevated resistance in these rural areas highlights the need for targeted antimicrobial stewardship and improved diagnostic support.

Isolates from canine patients residing in counties with high median household incomes showed higher odds of resistance to select antimicrobials, including amoxicillin, amoxicillin–clavulanic acid, and the third-generation cephalosporin cefpodoxime, compared with those from lower-middle-income counties. This pattern may reflect differences in veterinary care–seeking behavior and antimicrobial exposure, as pet owners in high-income areas may be more likely to access frequent veterinary care and receive more antimicrobial prescriptions, including cefpodoxime, which is classified by the World Health Organization as a critically important antimicrobial and is considered essential for treating serious human infections. Another possible explanation for this pattern could be sampling bias: highly specialized veterinary clinics may see more complicated or refractory cases and be more likely to be in high-income counties. These findings warrant ongoing monitoring, and targeted antimicrobial stewardship interventions which are critical to ensure judicious use of these agents and to mitigate potential risks across the One Health spectrum, encompassing human, animal, and environmental health [30]. In contrast, isolates from canine patients residing in counties with lower -middle median household incomes have lower odds of resistance, which could be driven by reduced access to veterinary care, less diagnostic testing, or cost-related care decisions. Consistent with this interpretation, previous research has documented substantial regional variation in veterinary diagnostic testing in the United States, highlighting inequities in access to and uptake of veterinary care that may contribute to observed differences in resistance patterns across regions [31]. Collectively, these findings emphasize the complex interplay between socioeconomic conditions, access to veterinary healthcare, diagnostic practices, and antimicrobial use, underscoring the need to tailor antimicrobial stewardship strategies to the socioeconomic realities of different communities in the United States.

The inverse relationship between the percentage of dogs with positive cultures for urinary *E. coli* and AST results each year relative to the county-level dog population and the odds of AMR suggests that greater use of veterinary and diagnostic services may support testing of first-occurrence cases and reduce inappropriate antimicrobial use. Frequent culture submission enables empirical therapy to be refined based on susceptibility results, potentially leading to shorter courses, narrower-spectrum drugs, and lower selection pressure, which aligns with antimicrobial stewardship principles [28]. However, it is also possible that higher numbers of cultures reflect repeated testing of refractory or complicated cases rather than first-occurrence infections, which could have artificially inflated the odds of AMR at the county level.

States with 1,500–1,999 housing units per veterinarian (lower veterinarian density) had higher odds of resistance to all antimicrobials compared with states with 500–999 units (higher veterinarian density). In areas with fewer veterinarians, empirical antimicrobial therapy may be more common, with prolonged or repeated courses administered without culture confirmation, potentially increasing selection pressure. Alternatively, testing may be concentrated on refractory or complicated cases, which could bias the observed odds of detecting resistance upward. These findings underscore the critical role of veterinarians in antimicrobial stewardship and highlight the need to improve access to veterinary care amid ongoing shortages of veterinarians [32].

Submission bias is a known issue with microbiological datasets like the one used in the present study, as culture and AST are not routinely performed for all clinical cases. Furthermore, as these data arise from passive surveillance, they may not be fully representative and are susceptible to potential selection bias. Consequently, the dataset likely reflects an overrepresentation of urinary *E. coli* isolates cultured from patients with recurrent, severe, or chronic infections, rather than from the broader population of infected canine patients in the United States. This selective testing pattern may result in an overrepresentation of resistant isolates and, consequently, an overestimation of the true prevalence of AMR in urinary *E. coli* from dogs. In contrast, mild infections, where isolates are more likely to be susceptible, may be underrepresented. Therefore, these findings should be interpreted with caution, acknowledging that submission bias may have skewed resistance estimates upward relative to the general clinical population. Another limitation is the laboratory use of outdated Clinical and Laboratory Standards breakpoints for interpretation, which have been significantly revised, particularly for fluoroquinolones. Compared with newer CLSI criteria [33], which define resistance at substantially lower minimum inhibitory concentration thresholds (e.g., ≥ 0.5 µg/mL versus ≥ 4.0 µg/mL in our dataset). This approach likely results in a systematic underestimation of fluoroquinolone resistance. Consequently, the fluoroquinolone resistance reported in this study should be interpreted as conservative estimates. Furthermore, the zone diameter measurements were not available in the dataset. Although this study benefits from seven years of comprehensive national AST data, the lack of detailed patient-level antimicrobial usage histories limits causal inference. Decisions to perform culture and AST are made on a case-by-case basis, often influenced by clinical severity and financial considerations, which may not be fully captured by county-level median household income. Many clients may decline testing due to cost, opting for empiric broad-spectrum therapy and only pursuing culture if empirical treatment fails. Additionally, there is potential misclassification of infection episodes (e.g., inability to distinguish incident from recurrent infections), and the use of proxy variables such as household median income, veterinarian density, and estimation of county-level dog population, which is based on national statistics. Furthermore, follow-up cultures collected during or after treatment could not be distinguished from the dataset. Future studies should integrate detailed patient-level data, including history of antimicrobial exposure, comorbidities, and treatment history, to better control for confounding. Prospective cohort studies and the incorporation of finer-scale socioeconomic and geographic variables would further enhance understanding of AMR dynamics of urinary *E. coli* isolates from canine patients in the United States.

## Conclusions

In conclusion, patient-level factors, particularly recent repeated positive cultures, are associated with AMR in canine urinary *E. coli*, likely reflecting cumulative antimicrobial exposure and/or chronic resistant infections. These findings suggest that patient history is a critical determinant when assessing AMR risk. Additionally, lower odds of AMR were observed in counties with higher microbiological testing frequency and in states with greater veterinarian density, which may reflect differences in access to veterinary care or diagnostic practices. However, as these variables are measured at the group level, they do not capture individual-level veterinary care. Future research incorporating patient-level measures of care access and prescribing practices will be crucial in this regard.

## Supplementary Information

Supplementary Material 1.

## Data Availability

The datasets used and/or analysed during the current study are available from the corresponding author on reasonable request.

## Abbreviations

AMR: Antimicrobial resistance
AST: Antimicrobial susceptibility testing
DAG: Directed acyclic graph
NNPC6: Number of prior positive cultures from the last six months
