# Supplementary material for: Exploratory analysis of risk factors associated with antimicrobial resistance in urinary Escherichia coli from dogs in the United States (2018–2024)
Source: BMC Vet Res. 2026 Jun 24;22:457. doi: 10.1186/s12917-026-05659-6 (PMC13445815; doi:10.1186/s12917-026-05659-6)
Supplement: Supplementary file 1 [file 12917_2026_5659_MOESM1_ESM.docx]

**Supporting information**

**Supplementary Figure 1**. Flowchart showing the frequency of individual and simultaneous antimicrobial susceptibility testing of urinary *E. coli* from dogs (2018 – 2024)

**Supplementary Table 1**. Description of variables included in the multilevel analysis of antimicrobial resistance in canine urinary *E. coli*

| **Variable name** | **Description** | **Hierarchical level** | **How it was calculated** | **Data source** |
| --- | --- | --- | --- | --- |
| Month | Calendar month of sample submission | Patient | Extracted from the sample submission date | IDEXX laboratory dataset |
| Year | Calendar year of sample submission | Patient | Extracted from the sample submission date | IDEXX laboratory dataset |
| Age group | Age of dogs categorized into defined groups (e.g., ≤1, 2–6, 7–11, ≥12 years) | Patient | Derived from reported age at sample submission | IDEXX laboratory dataset |
| Sex | Biological sex of the dog (male, female, unknown) | Patient | Reported by the submitting veterinarian | IDEXX laboratory dataset |
| Isolates from patients with at least one prior positive culture in the last 6 months | An indicator of whether a dog had ≥1 positive culture within the preceding 6 months | Patient | Based on the unique patient ID and submission history | IDEXX laboratory dataset |
| Number of prior positive cultures in the last 6 months | Count of positive cultures per dog within the preceding 6 months | Patient | Based on the unique patient ID and submission history | IDEXX laboratory dataset |
| Median household income | Median income of households in the county of the submitting veterinary clinic | County | Linked to the veterinary clinic’s county FIPS code | USDA, 2024 |
| Rural-Urban Continuum Codes | Rural–Urban Commuting Area code of the submitting clinic’s county | County | Linked to the county FIPS code in the IDEXX lab dataset | USDA, 2025 |
| Percentage tested | The percentage of dogs with positive culture each year relative to the estimated population of dogs in the county | County | Calculated as (# dogs with positive culture each year / estimated population of dogs in the county) × 100 | IDEXX laboratory dataset |
| Number of housing units per veterinarian | Ratio of housing units to the number of veterinarians in each state | State | Linked to the state where the submitting veterinary clinic is | Ouedraogo et al., 2019 |

**Supplementary Table 2**. Minimum inhibitory concentrations reported in the microbiological dataset used to classify urinary *E. coli* isolates from dogs into susceptible, intermediate, and resistant categories (2018–2024)

|  | **MICs reported (µg/mL)** | | |
| --- | --- | --- | --- |
| **Antimicrobial** | **Susceptible** | **Intermediate** | **Resistance** |
| AMOXI | ≤8 | 16 | ≥32 |
| AMOCLA | ≤8/4 | 16/8 | ≥32/16 |
| TRISUL | ≤2/38 | not defined | ≥4/76 |
| CEFOV | ≤2 | 4 | ≥8 |
| CEFPOD | ≤2 | 4 | ≥8 |
| ENROF | ≤0.12 | 1-2 | ≥4 |
| MARBOF | ≤0.12 | 1-2 | ≥4 |

AMOXI-Amoxicillin, AMOCLA-Amoxicillin clavulanic acid, TRISUL-Trimethoprim-sulfamethoxazole, CEFOV-Cefovecin, CEFPOD-Cefpodoxime, ENROF-Enrofloxacin, MARBOF-Marbofloxacin

**Supplementary Table 3**. Antimicrobial susceptibility profiles of urinary *E. coli* isolates from dogs in the USA (2018 – 2024)

| Antimicrobial class | Penicillin | | | | | | Potentiated sulfonamide | | |
| --- | --- | --- | --- | --- | --- | --- | --- | --- | --- |
| Antimicrobial | **AMOXI** | | | **AMOCLA** | | | **TRISUL** | | |
|  | **S** | **I** | **R** | **S** | **I** | **R** | **S** | **I** | **R** |
| n (%) | 265,546 (67.40) | 20,528 (5.21) | 107,898 (27.39) | 319,617 (81.13) | 11,346 (2.88) | 63,009(15.99) | 365,855 (92.86) | 30  (0.01) | 28,087 (7.13) |
| 95% CI | 67.26 – 67.55 | 5.14 – 5.28 | 27.25 – 27.53 | 78.71 – 78.87 | 2.83 – 2.93 | 15.88 – 16.11 | 92.78 – 92.94 | 0.01 – 0.01 | 7.05 – 7.21 |

| Cephalosporins (3rd gen.) | | | | | | Fluoroquinolones | | | | | |
| --- | --- | --- | --- | --- | --- | --- | --- | --- | --- | --- | --- |
| **CEFOV** | | | **CEFPOD** | | | **ENROF** | | | **MARBOF** | | |
| **S** | **I** | **R** | **S** | **I** | **R** | **S** | **I** | **R** | **S** | **I** | **R** |
| 329,457 (83.62) | 3,889  (0.99) | 60,626 (15.39) | 333,776 (84.72) | 326 (0.08) | 59,870 (15.20) | 348,990 (88.58) | 11,002 (2.79) | 33,980 (8.62) | 359,419 (91.23) | 1,283 (0.33) | 33,270 (8.44) |
| 83.51 – 83.74 | 0.95 – 1.02 | 15.28 – 15.50 | 84.61 – 84.83 | 0.07 – 0.09 | 15.08 – 15.31 | 88.48 – 88.68 | 2.74 – 2.84 | 8.53 – 8.71 | 91.14 – 91.31 | 0.31 – 0.34 | 8.36 – 8.53 |

AMOXI-Amoxicillin, AMOCLA-Amoxicillin clavulanic acid, TRISUL-Trimethoprim-sulfamethoxazole, CEFOV-Cefovecin, CEFPOD-Cefpodoxime, ENROF-Enrofloxacin, MARBOF-Marbofloxacin, S-Susceptible, I-Intermediate, R-Resistant, n – number of isolates, CI- Confidence Interval

**Supplementary Table 4**. Univariable analysis of the association between antimicrobial resistance and independent variables for urinary *E. coli* isolates from dogs in the USA (2018–2024)

|  |  | **AMOXI** | | | **AMOCLA** | | | **TRISUL** | | |
| --- | --- | --- | --- | --- | --- | --- | --- | --- | --- | --- |
| **Predictor** | **Category** | **OR (95% CI)** | **p-value** | **PLR** | **OR (95% CI)** | **p-value** | **PLR** | **OR (95% CI)** | **p-value** | **PLR** |
| Month | January (Ref.) | - | - | **< 0.001** | - | - | **< 0.001** | - | - | 0.913 |
|  | February | 0.99 (9.95 – 1.03) | 0.755 |  | 1.01 (0.97 – 1.06) | 0.702 |  | 1.01 (0.94 – 1.08) | 0.831 |  |
|  | March | 0.99 (9.95 – 1.03) | 0.515 |  | 1.00 (0.95 – 1.05) | 0.947 |  | 1.02 (0.95 – 1.09) | 0.581 |  |
|  | April | 0.99 (0.95 – 1.03) | 0.533 |  | 0.99 (0.94 – 1.04) | 0.652 |  | 0.99 (0.93 – 1.07) | 0.928 |  |
|  | May | 0.97 (0.93 – 1.01) | 0.123 |  | 0.97 (0.93 – 1.02) | 0.162 |  | 0.97 (0.90 – 1.04) | 0.384 |  |
|  | June | **0.94 (0.91 – 0.98)** | **0.003** |  | 0.97 (0.92 – 1.02) | 0.125 |  | 0.97 (0.91 – 1.04) | 0.315 |  |
|  | July | **0.93 (0.89 – 0.96)** | **<0.001** |  | 0.96 (0.92 – 1.01) | 0.077 |  | 0.95 (0.89 – 1.02) | 0.128 |  |
|  | August | 0.98 (0.94 – 1.02) | 0.200 |  | 1.00 (0.96 – 1.04) | 0.859 |  | 0.98 (0.92 – 1.05) | 0.516 |  |
|  | September | 0.99 (0.95 – 1.03) | 0.479 |  | 1.02 (0.98 – 1.07) | 0.441 |  | 1.01 (0.94 – 1.08) | 0.780 |  |
|  | October | 0.98 (0.94 – 1.02) | 0.308 |  | 1.02 (0.97 – 1.06) | 0.509 |  | 1.03 (0.96 – 1.10) | 0.389 |  |
|  | November | 1.02 (0.98 – 1.06) | 0.391 |  | 1.03 (0.99 – 1.08) | 0.251 |  | 1.04 (0.97 – 1.12) | 0.261 |  |
|  | December | 1.02 (0.98 – 1.06) | 0.229 |  | 1.05 (1.01 – 1.10) | 0.022 |  | 1.04 (0.97 – 1.12) | 0.249 |  |
|  |  |  |  | **< 0.001** |  |  | **< 0.001** |  |  | **< 0.001** |
| Year |  | **0.98 (0.97 – 0.98)** | **< 0.001** |  | **0.97 (0.96 – 0.98)** |  |  | **0.95 (0.94 – 0.96)** | **< 0.001** |  |
|  |  |  |  | **< 0.001** |  |  | **< 0.001** |  |  | **< 0.001** |
| Age group | Adult (2 - 6 years) (Ref.) | - | - |  | - | - |  | - | - |  |
|  | Geriatric (12+) | **1.35 (1.32 – 1.38)** | **< 0.001** |  | **1.27 (1.24 – 1.31)** | **< 0.001** |  | **1.22 (1.17 – 1.27)** | **< 0.001** |  |
|  | Puppy/Juvenile (0–1) | **0.68 (0.66 – 0.70)** | **< 0.001** |  | **0.66 (0.64 – 0.69)** | **< 0.001** |  | **0.91 (0.86 – 0.97)** | **< 0.001** |  |
|  | Senior (7–11) | **1.21 (1.18 – 1.24)** | **< 0.001** |  | **1.13 (1.10 – 1.16)** | **< 0.001** |  | **1.13 (1.09 – 1.18)** | **< 0.001** |  |
|  |  |  |  | 0.090 |  |  | **0.003** |  |  | 1.000 |
| Median household income | High (≥ $100k) (Ref.) | - | - |  | - | - |  | - | - |  |
|  | Low (< $40k) | 1.11 (0.72 – 1.72) | 0.653 |  | 0.96 (0.58 – 1.59) | 0.889 |  | 0.70 (0.31 – 1.57) | 0.405 |  |
|  | Lower-Middle ($40k < $70k) | 0.96 (0.90 – 1.02) | 0.116 |  | **0.91 (0.85 – 0.98)** | **0.0145** |  | 0.98 (0.89 – 1.08) | 0.701 |  |
|  | Upper-Middle ($70k < $100k) | 0.99 (0.94 – 1.04) | 0.734 |  | 0.98 (0.91 – 1.05) | 0.516 |  | 0.98 (0.89 – 1.07) | 0.637 |  |
|  |  |  |  | **0.028** |  |  | **< 0.001** |  |  | **0.022** |
| Rural-Urban Continuum Codes | 1 (Ref.) |  |  |  |  |  |  |  |  |  |
|  | 2 | 1.00 (0.96 – 1.04) | 0.956 |  | **0.98 (0.93 – 1.04)** | **0.379** |  | 0.98 (0.91 – 1.05) | 0.501 |  |
|  | 3 | **0.93 (0.88 – 0.98)** | **0.005** |  | **0.89 (0.83 – 0.95)** | **< 0.001** |  | 0.98 (0.90 – 1.07) | 0.673 |  |
|  | 4 | 0.99 (0.93 – 1.06) | 0.772 |  | **0.92 (0.85 – 0.99)** | **0.037** |  | 1.07 (0.95 – 1.20) | 0.283 |  |
|  | 5 | 1.01 (0.89 – 1.15) | 0.848 |  | 0.98 (0.83 – 1.15) | 0.824 |  | 1.07 (0.85 – 1.35) | 0.565 |  |
|  | 6 | 0.95 (0.88 – 1.03) | 0.209 |  | **0.88 (0.80 – 0.96)** | **0.017** |  | 1.09 (0.95 – 1.25) | 0.237 |  |
|  | 7 | 0.95 (0.85 – 1.06) | 0.324 |  | **0.91 (0.79 – 1.04)** | 0.157 |  | **1.31 (1.08 – 1.59)** | **0.005** |  |
|  | 8 | 0.93 (0.82 – 1.06) | 0.280 |  | **0.80 (0.68 – 0.94)** | **0.007** |  | 0.89 (0.70 – 1.13) | 0.338 |  |
|  | 9 | 0.85 (0.71 – 1.02) | 0.079 |  | **0.70 (0.55 – 0.89)** | **0.004** |  | 0.92 (0.65 – 1.31) | 0.631 |  |
|  |  |  |  | 0.412 |  |  | **< 0.001** |  |  | **< 0.001** |
| Sex | Female (Ref.) |  |  |  |  |  |  |  |  |  |
|  | Male | 0.99 (0.97 – 1.01) | 0.480 |  | **0.96 (0.94 – 0.99)** | **< 0.001** |  | **1.08 (1.05 – 1.12)** | **< 0.001** |  |
|  |  |  |  | **0.002** |  |  | **0.003** |  |  | 1.000 |
| Number of housing units per veterinarian | 2000 - 3000 (Ref.) |  |  |  |  |  |  |  |  |  |
|  | 1000 - 1499 | 0.96 (0.71 – 1.29) | 0.787 |  | 0.91 (0.62 – 1.35) | 0.638 |  | 0.91 (0.52 – 1.61) | 0.750 |  |
|  | 1500 - 1999 | 1.05 (0.78 – 1.43) | 0.760 |  | 1.01 (0.68 – 1.50) | 0.953 |  | 1.11 (0.62 – 1.99) | 0.732 |  |
|  | 500 - 999 | 0.79 (0.58 – 1.08) | 0.134 |  | 0.71 (0.47 – 1.05) | 0.094 |  | 0.70 (0.39 – 1.27) | 0.239 |  |
|  |  |  |  | **< 0.001** |  |  | **< 0.001** |  |  | **< 0.001** |
| Percentage tested (%) |  | **0.99 (0.99 – 0.99)** | **< 0.001** |  | **0.99 (0.99 – 0.99)** | **< 0.001** |  | **0.98 (0.98 – 0.99)** | **< 0.001** |  |
|  |  |  |  | **< 0.001** |  |  | **< 0.001** |  |  | **< 0.001** |
|  | Isolates from patients without prior positive culture in the last 6 months (Ref.) |  |  |  |  |  |  |  |  |  |
| Isolates from patients with at least one prior positive culture in the last 6 months |  | **1.37 (1.33 – 1.40)** | **< 0.001** |  | **1.36 (1.32 – 1.40)** | **< 0.001** |  | **1.85 (1.78 – 1.92)** | **< 0.001** |  |
|  |  |  |  | **< 0.001** |  |  | **< 0.001** |  |  | **< 0.001** |
|  | Isolates from patients without prior positive culture in the last 6 months (Ref.) |  |  |  |  |  |  |  |  |  |
| Number of prior positive cultures in the last 6 months | 1 | **1.29 (1.26 – 1.33)** | **< 0.001** |  | **1.28 (1.24 – 1.32)** | **< 0.001** |  | **1.70 (1.63 – 1.77)** | **< 0.001** |  |
|  | 2 | **1.72 (1.62 – 1.83)** | **< 0.001** |  | **1.71 (1.60 – 1.82)** | **< 0.001** |  | **2.51 (2.31 – 2.72)** | **< 0.001** |  |
|  | 3 | **2.39 (2.11 – 2.71)** | **< 0.001** |  | **2.27 (1.99 – 2.59)** | **< 0.001** |  | **3.17 (2.69 – 3.72)** | **< 0.001** |  |
|  | 4 | **2.97 (2.25 – 3.93)** | **< 0.001** |  | **2.71 (2.07 – 3.55)** | **< 0.001** |  | **3.97 (2.88 – 5.49)** | **< 0.001** |  |
|  | 5 | **6.52 (3.17 – 13.40)** | **< 0.001** |  | **2.85 (1.54 – 5.27)** | **< 0.001** |  | **3.39 (1.65 – 7.00)** | **< 0.001** |  |
|  |  |  |  |  |  |  |  |  |  |  |

|  |  | **CEFOV** | | | **CEFPOD** | | | **ENROF** | | |
| --- | --- | --- | --- | --- | --- | --- | --- | --- | --- | --- |
| **Predictor** | **Category** | **OR (95% CI)** | **p-value** | **PLR** | **OR (95% CI)** | **p-value** | **PLR** | **OR (95% CI)** | **p-value** | **PLR** |
| Month | January (Ref.) | - | - | **< 0.001** | - | - | **0.002** | - | - | **< 0.001** |
|  | February | 0.99 (0.94 – 1.04) | 0.634 |  | 1.00 (0.95 – 1.05) | 0.997 |  | 0.95 (0.90 – 1.01) | 0.128 |  |
|  | March | 0.99 (0.95 – 1.04) | 0.760 |  | 1.01 (0.96 – 1.06) | 0.695 |  | 0.98 (0.92 – 1.04) | 0.439 |  |
|  | April | 0.96 (0.92 – 1.01) | 0.132 |  | 0.99 (0.94 – 1.04) | 0.627 |  | 0.95 (0.89 – 1.01) | 0.100 |  |
|  | May | **0.96 (0.92 – 1.01)** | **0.010** |  | 0.98 (0.93 – 1.03) | 0.504 |  | **0.900 (0.85 – 0.96)** | **< 0.001** |  |
|  | June | **0.95 (0.90 – 0.99)** | **0.022** |  | 0.99 (0.94 – 1.04) | 0.559 |  | **0.87 (0.82 – 0.92)** | **< 0.001** |  |
|  | July | 0.97 (0.92 – 1.01) | 0.155 |  | 0.99 (0.94 – 1.04) | 0.582 |  | **0.89 (0.82 – 0.92)** | **< 0.001** |  |
|  | August | 0.99 (0.95 – 1.04) | 0.752 |  | 1.02 (0.97 – 1.07) | 0.354 |  | **0.89 (0.84 – 0.94)** | **< 0.001** |  |
|  | September | 1.01 (0.97 – 1.06) | 0.550 |  | 1.04 (0.99 – 1.09) | 0.150 |  | 0.96 (0.91 – 1.02) | 0.225 |  |
|  | October | 1.00 (0.96 – 1.05) | 0.959 |  | 1.02 (0.97 – 1.07) | 0.352 |  | 1.00 (0.95 – 1.06) | 0.714 |  |
|  | November | 1.01 (0.96 – 1.06) | 0.734 |  | 1.04 (0.99 – 1.09) | 0.133 |  | 1.00 (0.95 – 1.07) | 0.895 |  |
|  | December | 1.04 (1.00 – 1.09) | 0.084 |  | **1.06 (1.01 – 1.12)** | **0.014** |  | 1.01 (0.95 – 1.07) | 0.772 |  |
|  |  |  |  | **< 0.001** |  |  | **< 0.001** |  |  | **< 0.001** |
| Year |  | **0.98 (0.97 – 0.98)** | **< 0.001** |  | **0.99 (0.99 – 1.00)** | **< 0.001** |  | **0.96 (0.95 – 0.97)** | **< 0.001** |  |
|  |  |  |  | **< 0.001** |  |  | **< 0.001** |  |  | **< 0.001** |
| Age group | Adult (2 - 6 years) (Ref.) | - | - |  | - | - |  | - | - |  |
|  | Geriatric (12+) | **1.33 (1.29 – 1.37)** | **< 0.001** |  | **1.27 (1.23 – 1.31)** | **< 0.001** |  | **1.58 (1.52–1.64)** | **< 0.001** |  |
|  | Puppy/Juvenile (0–1) | **0.61 (0.59 – 0.64)** | **< 0.001** |  | **0.64 (0.61 – 0.67)** | **< 0.001** |  | **0.49 (0.46–0.52)** | **< 0.001** |  |
|  | Senior (7–11) | **1.17 (1.34 – 1.20)** | **< 0.001** |  | **1.12 (1.09 – 1.16)** | **< 0.001** |  | **1.34 (1.29–1.39)** | **< 0.001** |  |
|  |  |  |  | **0.012** |  |  | **< 0.001** |  |  | **<0.001** |
| Median household income | High (≥ $100k) (Ref.) | - | - |  | - | - |  | - | - |  |
|  | Low (< $40k) | 1.07 (0.63 – 1.81) | 0.796 |  | 1.02 (0.59 – 1.76) | 0.936 |  | 0.60 (0.27–1.30) | 0.195 |  |
|  | Lower-Middle ($40k < $70k) | 0.93 (0.86 – 1.00) | 0.051 |  | **0.89 (0.82 – 0.97)** | **0.006** |  | 0.97 (0.86–1.09) | 0.604 |  |
|  | Upper-Middle ($70k < $100k) | 0.98 (0.91 – 1.05) | 0.575 |  | 0.97 (0.90 – 1.04) | 0.461 |  | 0.98 (0.88–1.10) | 0.757 |  |
|  |  |  |  | **< 0.001** |  |  | **< 0.001** |  |  | **<0.001** |
| Rural-Urban Continuum Codes | 1 (Ref.) |  |  |  |  |  |  |  |  |  |
|  | 2 | 0.98 (0.93 – 1.04) | 0.524 |  | 0.97 (0.91 – 1.03) | 0.310 |  | 1.02 (0.93–1.10) | 0.744 |  |
|  | 3 | **0.91 (0.85 – 0.97)** | **0.006** |  | **0.89 (0.83 – 0.96)** | **0.002** |  | 0.95 (0.86–1.05) | 0.297 |  |
|  | 4 | 0.96 (0.88 – 1.04) | 0.306 |  | 0.93 (0.84 – 1.03) | 0.107 |  | 0.98 (0.86–1.11) | 0.717 |  |
|  | 5 | 0.95 (0.80 – 1.12) | 0.546 |  | 0.89 (0.74 – 1.07) | 0.195 |  | 1.15 (0.91–1.45) | 0.252 |  |
|  | 6 | 0.91 (0.82 – 1.01) | 0.060 |  | **0.87 (0.78 – 0.97)** | **0.010** |  | 1.02 (0.88–1.17) | 0.807 |  |
|  | 7 | 0.96 (0.83 – 1.10) | 0.556 |  | 0.90 (0.77 – 1.05) | 0.192 |  | 0.98 (0.80–1.20) | 0.829 |  |
|  | 8 | **0.83 (0.70 – 0.99)** | **0.035** |  | **0.81 (0.68 – 0.97)** | **0.024** |  | 0.89 (0.70–1.12) | 0.317 |  |
|  | 9 | 0.85 (0.67 – 1.09) | 0.201 |  | **0.77 (0.59 – 1.00)** | **0.045** |  | 0.89 (0.64–1.24) | 0.498 |  |
|  |  |  |  | **< 0.001** |  |  | **< 0.001** |  |  | **0.014** |
| Sex | Female (Ref.) |  |  |  |  |  |  |  |  |  |
|  | Male | **0.92 (0.90 – 0.94)** | **< 0.001** |  | **0.92 (0.90 – 0.95)** | **< 0.001** |  | **1.04 (1.01–1.07)** | **0.008** |  |
|  |  |  |  | **0.005** |  |  | **0.003** |  |  | **0.005** |
| Number of housing units per veterinarian | 2000 - 3000 (Ref.) |  |  |  |  |  |  |  |  |  |
|  | 1000 - 1499 | 0.85 (0.55 – 1.32) | 0.477 |  | 0.88 (0.56 – 1.38) | 0.587 |  | 0.76 (0.38–1.50) | 0.428 |  |
|  | 1500 - 1999 | 0.95 (0.60 – 1.49) | 0.819 |  | 0.97 (0.60 – 1.56) | 0.908 |  | 0.96 (0.47–1.95) | 0.903 |  |
|  | 500 - 999 | 0.64 (0.40 – 1.02) | 0.059 |  | 0.64 (0.38 – 1.02) | 0.067 |  | 0.53 (0.26–1.10) | 0.089 |  |
|  |  |  |  | **< 0.001** |  |  | **< 0.001** |  |  | **< 0.001** |
| Percentage tested (%) |  | **0.99 (0.99 – 0.99)** | **< 0.001** |  | **0.99 (0.99 – 1.00)** | **< 0.001** |  | **0.98 (0.98 – 0.99)** | **< 0.001** |  |
|  |  |  |  | **< 0.001** |  |  | **< 0.001** |  |  | **< 0.001** |
|  | Isolates from patients without prior positive culture in the last 6 months (Ref.) |  |  |  |  |  |  |  |  |  |
| Isolates from patients with at least one prior positive culture in the last 6 months |  | **1.44 (1.40 – 1.48)** | **< 0.001** |  | **1.37 (1.33 – 1.41)** | **< 0.001** |  | **2.28 (2.20–2.36)** | **< 0.001** |  |
|  |  |  |  | **< 0.001** |  |  | **< 0.001** |  |  | **< 0.001** |
|  |  |  |  |  |  |  |  |  |  |  |
| Number of prior positive cultures in the last 6 months | 1 | **1.34 (1.30 – 1.38)** | **< 0.001** |  | **1.28 (1.24 – 1.32)** | **< 0.001** |  | **2.05 (1.98–2.13)** | **< 0.001** |  |
|  | 2 | **1.88 (1.76 – 2.01)** | **< 0.001** |  | **1.75 (1.64 – 1.87)** | **< 0.001** |  | **3.44 (3.20–3.70)** | **< 0.001** |  |
|  | 3 | **2.63 (2.30 – 3.00)** | **< 0.001** |  | **2.40 (2.09 – 2.75)** | **< 0.001** |  | **5.04 (4.34–5.85)** | **< 0.001** |  |
|  | 4 | **2.82 (2.15 – 3.71)** | **< 0.001** |  | **2.38 (1.80 – 3.15)** | **< 0.001** |  | **7.19 (5.25–9.83)** | **< 0.001** |  |
|  | 5 | **3.07 (1.64 – 5.77)** | **< 0.001** |  | **2.71 (1.43 – 5.15)** | **0.002** |  | **8.47 (4.02–17.83)** | **< 0.001** |  |

|  |  | **MARBOF** | | |
| --- | --- | --- | --- | --- |
| **Predictor** | **Category** | **OR (95% CI)** | **p-value** | **PLR** |
| Month | January (Ref.) | - | - | **< 0.001** |
|  | February | **0.92 (0.86–0.99)** | **0.033** |  |
|  | March | 0.97 (0.91–1.04) | 0.430 |  |
|  | April | **0.93 (0.86–0.99)** | **0.045** |  |
|  | May | **0.88 (0.82–0.94)** | **< 0.001** |  |
|  | June | **0.86 (0.80–0.92)** | **< 0.001** |  |
|  | July | **0.89 (0.83–0.95)** | **< 0.001** |  |
|  | August | **0.88 (0.82–0.94)** | **< 0.001** |  |
|  | September | 0.98 (0.92–1.05) | 0.619 |  |
|  | October | 0.99 (0.93–1.05) | 0.708 |  |
|  | November | 1.01 (0.94–1.08) | 0.723 |  |
|  | December | 0.98 (0.92–1.05) | 0.557 |  |
|  |  |  |  | **< 0.001** |
| Year |  | **0.93 (0.92–0.94)** | **< 0.001** |  |
|  |  |  |  | **< 0.001** |
| Age group | Adult (2 - 6 years) (Ref.) | - | - |  |
|  | Geriatric (12+) | **1.75 (1.67–1.84)** | **< 0.001** |  |
|  | Puppy/Juvenile (0–1) | **0.32 (0.29–0.34)** | **< 0.001** |  |
|  | Senior (7–11) | **1.44 (1.37-1.51)** | **< 0.001** |  |
|  |  |  |  | 0.535 |
| Median household income | High (≥ $100k) (Ref.) | - | - |  |
|  | Low (< $40k) | 0.60 (0.24–1.51) | 0.272 |  |
|  | Lower-Middle ($40k < $70k) | 1.00 (0.87–1.15) | 0.978 |  |
|  | Upper-Middle ($70k < $100k) | 0.99 (0.86-1.14) | 0.965 |  |
|  |  |  |  | 0.296 |
| Rural-Urban Continuum Codes | 1 (Ref.) |  |  |  |
|  | 2 | 1.02 (0.92 – 1.33) | 0.733 |  |
|  | 3 | 0.95 (0.84 – 1.07) | 0.381 |  |
|  | 4 | 1.00 (0.86 – 1.16) | 0.957 |  |
|  | 5 | 1.16 (0.87 – 1.54) | 0.309 |  |
|  | 6 | 1.02 (0.86 – 1.20) | 0.829 |  |
|  | 7 | 1.08 (0.85 – 1.38) | 0.501 |  |
|  | 8 | 0.78 (0.58 – 1.04) | 0.094 |  |
|  | 9 | 0.97 (0.66 – 1.42) | 0.867 |  |
|  |  |  |  | **< 0.001** |
| Sex | Female (Ref.) |  |  |  |
|  | Male | **1.08 (1.04 – 1.12)** | **< 0.001** |  |
|  |  |  |  | **0.005** |
| Number of housing units per veterinarian | 2000 - 3000 (Ref.) |  |  |  |
|  | 1000 - 1499 | 0.68 (0.29 – 1.59) | 0.372 |  |
|  | 1500 - 1999 | 0.87 (0.36 – 2.09) | 0.753 |  |
|  | 500 - 999 | 0.44 (0.18 – 1.08) | 0.072 |  |
|  |  |  |  | **< 0.001** |
| Percentage tested (%) |  | **0.98 (0.98–0.99)** | **< 0.001** |  |
|  |  |  |  | **< 0.001** |
|  | Isolates from patients without prior positive culture in the last 6 months (Ref.) |  |  |  |
| Isolates from patients with at least one prior positive culture in the last 6 months |  | **2.34 (2.25 – 2.43)** | **< 0.001** |  |
|  |  |  |  | **< 0.001** |
|  | Isolates from patients without prior positive culture in the last 6 months (Ref.) |  |  |  |
| Number of prior positive cultures in the last 6 months | 1 | **2.10 (2.02–2.19)** | **< 0.001** |  |
|  | 2 | **3.49 (3.22–3.79)** | **< 0.001** |  |
|  | 3 | **5.44 (4.64–6.38)** | **< 0.001** |  |
|  | 4 | **6.40 (4.61–8.87)** | **< 0.001** |  |
|  | 5 | **12.39 (5.74–26.72)** | **< 0.001** |  |

Each model has three levels of random effects at the state, county, and patient levels

AMOXI-Amoxicillin, AMOCLA-Amoxicillin clavulanic acid, TRISUL-Trimethoprim-sulfamethoxazole, CEFOV-Cefovecin, CEFPOD-Cefpodoxime, ENROF-Enrofloxacin, MARBOF-Marbofloxacin, PLR-Partial Likelihood Ratio Test. Inverse OR values (1/OR) aid interpretation when the reference category is reversed.

**Supplementary Table 5**. Pairwise comparison of the effect of prior positive cultures within the preceding six months on the antimicrobial resistance in urinary *E. coli* isolates from dogs in the USA (2018–2024)

|  |  | **AMOXI** | | | **AMOCLA** | | | **TRISUL** | | | **CEFOV** | | |
| --- | --- | --- | --- | --- | --- | --- | --- | --- | --- | --- | --- | --- | --- |
| Contrast | | OR | 95% CI (OR) | Adjusted p-value | OR | 95% CI (OR) | Adjusted p-value | OR | 95% CI (OR) | Adjusted p-value | OR | 95% CI (OR) | Adjusted p-value |
| 1 vs 0 | | **1.29** | **1.25 – 1.32** | **<0.001** | **1.27** | **1.23 – 1.31** | **<0.001** | **1.71** | **1.64 – 1.78** | **<0.001** | **1.33** | **1.29 – 1.38** | **<0.001** |
| 2 vs 0 | | **1.71** | **1.61 – 1.82** | **<0.001** | **1.70** | **1.59 – 1.82** | **<0.001** | **2.52** | **2.32 – 2.74** | **<0.001** | **1.87** | **1.75 – 2.00** | **<0.001** |
| 3 vs 0 | | **2.36** | **2.08 – 2.68** | **<0.001** | **2.25** | **1.97 – 2.56** | **<0.001** | **3.20** | **2.72 – 3.77** | **<0.001** | **2.60** | **2.27 – 2.97** | **<0.001** |
| 4 vs 0 | | **2.93** | **2.22 – 3.87** | **<0.001** | **2.69** | **2.05 – 3.53** | **<0.001** | **4.00** | **2.90 – 5.52** | **<0.001** | **2.78** | **2.14 – 3.61** | **<0.001** |
| 5 vs 0 | | **6.34** | **3.08 – 13.03** | **<0.001** | **2.78** | **1.51 – 5.10** | **0.014** | **3.42** | **1.66 – 7.08** | **0.011** | **2.97** | **1.58 – 5.58** | **0.009** |
| 2 vs 1 | | **1.33** | **1.25 – 1.41** | **<0.001** | **1.34** | **1.25 – 1.44** | **<0.001** | **1.47** | **1.34 – 1.62** | **<0.001** | **1.40** | **1.31 – 1.50** | **<0.001** |
| 3 vs 1 | | **1.85** | **1.64 – 2.13** | **<0.001** | **1.77** | **1.55 – 2.03** | **<0.001** | **1.87** | **1.58 – 2.21** | **<0.001** | **1.95** | **1.71 – 2.22** | **<0.001** |
| 4 vs 1 | | **2.27** | **1.69 – 3.05** | **<0.001** | **2.11** | **1.64 – 2.72** | **<0.001** | **2.34** | **1.70 – 3.23** | **<0.001** | **2.09** | **1.63 – 2.70** | **< 0.001** |
| 5 vs 1 | | **5.00** | **2.46 – 10.00** | **0.003** | 2.19 | 1.18 – 4.05 | 0.126 | 2.00 | 0.96 – 4.17 | 0.421 | 2.23 | 1.19 – 4.18 | 0.129 |
| 3 vs 2 | | **1.38** | **1.22 – 1.59** | **<0.001** | **1.33** | **1.15 – 1.55** | **0.001** | 1.27 | 0.99 – 1.63 | 0.088 | **1.39** | **1.21 – 1.60** | **0.001** |
| 4 vs 2 | | **1.71** | **1.30 – 2.27** | **0.002** | **1.58** | **1.20 – 2.08** | **0.015** | 1.59 | 0.99 – 2.55 | 0.066 | 1.49 | 1.13 – 1.97 | 0.060 |
| 5 vs 2 | | **3.67** | **1.79 – 7.69** | **0.005** | 1.64 | 0.89 – 3.02 | 0.620 | 1.36 | 0.66 – 2.82 | 0.964 | 1.59 | 0.84 – 3.01 | 0.711 |
| 4 vs 3 | | 1.23 | 0.92 – 1.67 | 0.712 | 1.19 | 0.89 – 1.58 | 0.845 | 1.25 | 0.88 – 1.78 | 0.803 | 1.07 | 0.80 – 1.43 | 0.998 |
| 5 vs 3 | | 2.68 | 1.31 – 5.56 | 0.086 | 1.23 | 0.74 – 2.05 | 0.986 | 1.07 | 0.51 – 2.23 | 1.000 | 1.14 | 0.60 – 2.16 | 0.999 |
| 5 vs 4 | | 2.17 | 1.00 – 4.76 | 0.349 | 1.03 | 0.54 – 1.95 | 1.000 | 0.85 | 0.39 – 1.85 | 0.999 | 1.07 | 0.54 – 2.13 | 1.000 |

|  |  | **CEFPOD** | | | **ENROF** | | | **MARBOF** | | |
| --- | --- | --- | --- | --- | --- | --- | --- | --- | --- | --- |
| Contrast | | OR | 95% CI (OR) | Adjusted p-value | OR | 95% CI (OR) | Adjusted p-value | OR | 95% CI (OR) | Adjusted p-value |
| 1 vs 0 | | **1.27** | **1.23** – **1.32** | **<0.001** | **2.06** | **1.98 – 2.15** | **<0.001** | **2.14** | **2.05 – 2.23** | **<0.001** |
| 2 vs 0 | | **1.74** | **1.62 – 1.87** | **<0.001** | **3.47** | **3.18 – 3.78** | **<0.001** | **3.57** | **3.29 – 3.89** | **<0.001** |
| 3 vs 0 | | **2.37** | **2.07 – 2.72** | **<0.001** | **5.07** | **4.27 – 6.01** | **<0.001** | **5.60** | **4.74 – 6.62** | **<0.001** |
| 4 vs 0 | | **2.34** | **1.77 – 3.08** | **<0.001** | **7.26** | **5.16 – 10.21** | **<0.001** | **6.60** | **4.84 – 9.00** | **<0.001** |
| 5 vs 0 | | **2.61** | **1.37 – 4.95** | **0.040** | **8.27** | **3.70 – 18.46** | **<0.001** | **12.38** | **5.79 – 26.50** | **<0.001** |
| 2 vs 1 | | **1.37** | **1.27 – 1.48** | **<0.001** | **1.68** | **1.56 – 1.82** | **<0.001** | **1.68** | **1.53 – 1.84** | **<0.001** |
| 3 vs 1 | | **1.86** | **1.61 – 2.14** | **<0.001** | **2.46** | **2.10 – 2.87** | **<0.001** | **2.62** | **2.16 – 3.17** | **<0.001** |
| 4 vs 1 | | **1.84** | **1.39 – 2.44** | **<0.001** | **3.52** | **2.57 – 4.83** | **<0.001** | **3.09** | **2.15 – 4.44** | **<0.001** |
| 5 vs 1 | | 2.05 | 1.08 – 3.88 | 0.243 | **4.01** | **1.91 – 8.41** | **0.003** | **5.79** | **2.73 – 12.27** | **<0.001** |
| 3 vs 2 | | **1.36** | **1.17 – 1.58** | **0.001** | **1.46** | **1.22 – 1.74** | **<0.001** | **1.56** | **1.33 – 1.83** | **<0.001** |
| 4 vs 2 | | 1.35 | 1.01 – 1.81 | 0.321 | **2.09** | **1.37 – 3.18** | **<0.001** | **1.84** | **1.19 – 2.85** | **0.004** |
| 5 vs 2 | | 1.50 | 0.79 – 2.83 | 0.820 | 2.38 | 0.82 – 6.93 | 0.196 | **3.45** | **1.62 – 7.35** | **0.018** |
| 4 vs 3 | | 0.99 | 0.73 – 1.35 | 1.000 | 1.43 | 0.84 – 2.43 | 0.287 | 1.18 | 0.72 – 1.94 | 0.939 |
| 5 vs 3 | | 1.10 | 0.57 – 2.12 | 0.999 | 1.63 | 0.76 – 3.49 | 0.795 | 2.21 | 0.82 – 5.98 | 0.333 |
| 5 vs 4 | | 1.11 | 0.56 – 2.19 | 0.999 | 1.14 | 0.46 – 2.81 | 0.998 | 1.87 | 0.66 – 5.27 | 0.644 |

Model adjusted for month, year, age, sex, age–sex interaction, Rural-Urban Continuum Codes, number of housing units per veterinarian, and median household income, with random effects at the state, county, and patient levels

AMOXI-Amoxicillin, AMOCLA-Amoxicillin clavulanic acid, TRISUL-Trimethoprim-sulfamethoxazole, CEFOV-Cefovecin, CEFPOD-Cefpodoxime, ENROF-Enrofloxacin, MARBOF-Marbofloxacin. Inverse OR values (1/OR) aid interpretation when the reference category is reversed.

**Supplementary Table 6**. Pairwise comparison of the interaction between age and sex on antimicrobial resistance in urinary *E. coli* isolates from dogs in the USA (2018–2024)

|  |  | **AMOXI** | | | **AMOCLA** | | | **TRISUL** | | |
| --- | --- | --- | --- | --- | --- | --- | --- | --- | --- | --- |
| Age Group | Contrast | OR | 95% CI (OR) | Adjusted p-value | OR | 95% CI (OR) | Adjusted p-value | OR | 95% CI (OR) | Adjusted p-value |
| Puppy/Juvenile (0-1yr) | Female - Male | **0.74** | **0.67 – 0.81** | **<0.001** | **0.69** | **0.62 – 0.76** | **<0.001** | **0.66** | **0.56 – 0.77** | **<0.001** |
| Adult (2-6yrs) | Female - Male | 0.98 | 0.93 – 1.04 | 0.726 | 0.97 | 0.91 – 1.03 | 0.525 | **0.85** | **0.77 – 0.93** | **0.003** |
| Senior (7-11yrs) | Female - Male | **1.12** | **1.09 – 1.15** | **<0.001** | **1.14** | **1.10 – 1.18** | **<0.001** | 0.97 | 0.92 – 1.03 | 0.538 |
| Geriatric (12yrs+) | Female - Male | **1.10** | **1.07 – 1.13** | **<0.001** | **1.16** | **1.12 – 1.20** | **<0.001** | 1.01 | 0.96 – 1.06 | 0.973 |

|  |  | **CEFOV** | | | **CEFPOD** | | | **ENROF** | | |
| --- | --- | --- | --- | --- | --- | --- | --- | --- | --- | --- |
| Age Group | Contrast | OR | 95% CI (OR) | Adjusted p-value | OR | 95% CI (OR) | Adjusted p-value | OR | 95% CI (OR) | Adjusted p-value |
| Puppy/Juvenile (0-1yr) | Female - Male | **0.68** | **0.60 – 0.77** | **<0.001** | **0.67** | **0.59 – 0.77** | **<0.001** | **0.66** | **0.56 – 0.77** | **<0.001** |
| Adult (2-6yrs) | Female - Male | 1.03 | 0.96 – 1.10 | 0.678 | 1.00 | 0.94 – 1.07 | 0.999 | 0.89 | 0.82 – 0.98 | 0.138 |
| Senior (7-11yrs) | Female - Male | **1.19** | **1.15 – 1.24** | **<0.001** | **1.18** | **1.14 – 1.23** | **<0.001** | **1.09** | **1.04 – 1.15** | **0.002** |
| Geriatric (12yrs+) | Female - Male | **1.22** | **1.18 – 1.27** | **<0.001** | **1.22** | **1.18 – 1.27** | **<0.001** | **1.09** | **1.04 – 1.15** | **<0.001** |

|  |  | **MARBOF** | | |
| --- | --- | --- | --- | --- |
| Age Group | Contrast | OR | 95% CI (OR) | Adjusted p-value |
| Puppy/Juvenile (0-1yr) | Female - Male | **0.44** | **0.35 – 0.55** | **<0.001** |
| Adult (2-6yrs) | Female - Male | **0.83** | **0.75 – 0.91** | **0.001** |
| Senior (7-11yrs) | Female - Male | **1.08** | **1.02 – 1.15** | **0.017** |
| Geriatric (12yrs+) | Female - Male | **1.09** | **1.03 – 1.15** | **0.001** |

The model did not adjust for any covariates but has random effects at the state, county, and patient levels

AMOXI-Amoxicillin, AMOCLA-Amoxicillin clavulanic acid, TRISUL-Trimethoprim-sulfamethoxazole, CEFOV-Cefovecin, CEFPOD-Cefpodoxime, ENROF-Enrofloxacin, MARBOF-Marbofloxacin. Inverse OR values (1/OR) aid interpretation when the reference category is reversed.

**Supplementary Table 7**. Full multivariable model showing the direct effects of all exposures of interest on antimicrobial resistance in urinary *E. coli* isolates from dogs in the USA

|  |  | **AMOXI** | | | | **AMOCLA** | | | |
| --- | --- | --- | --- | --- | --- | --- | --- | --- | --- |
| Predictor | Category | Estimates | Std. Error | OR (95% CI) | p-value | Estimates | Std. Error | OR (95% CI) | p-value |
| Intercept |  | -0.655 | 0.134 | - | < 0.001 | -1.295 | 0.179 | - | < 0.001 |
| Year |  | -0.023 | 0.002 | **0.98 (0.97 - 0.98)** | **< 0.001** | -0.029 | 0.002 | **0.97 (0.96 – 0.97)** | **< 0.001** |
| Month | January (Ref.) |  |  |  |  |  |  |  |  |
|  | February | -0.012 | 0.020 | 0.99 (0.95 - 1.03) | 0.533 | 0.000 | 0.024 | 1.00 (0.96 – 1.04) | 0.990 |
|  | March | -0.015 | 0.020 | 0.99 (0.95 - 1.02) | 0.430 | -0.007 | 0.023 | 0.99 (0.94 – 1.03) | 0.748 |
|  | April | -0.008 | 0.020 | 0.99 (0.95 – 1.03) | 0.705 | -0.009 | 0.024 | 0.99 (0.94 – 1.04) | 0.693 |
|  | May | -0.018 | 0.020 | 0.98 (0.94 – 1.02) | 0.354 | -0.026 | 0.024 | 0.97 (0.92 – 1.02) | 0.278 |
|  | June | -0.040 | 0.019 | **0.96 (0.93 – 0.99)** | **0.039** | -0.021 | 0.023 | 0.98 (0.94 – 1.03) | 0.359 |
|  | July | -0.051 | 0.019 | **0.95 (0.92 – 0.98)** | **0.008** | -0.026 | 0.023 | 0.97 (0.93 – 1.02) | 0.252 |
|  | August | -0.004 | 0.019 | 0.99 (0.96 – 1.03) | 0.836 | 0.011 | 0.023 | 1.01 (0.96 – 1.06) | 0.633 |
|  | September | 0.004 | 0.019 | 1.00 (0.97 – 1.04) | 0.819 | 0.030 | 0.023 | 1.03 (0.98 – 1.07) | 0.182 |
|  | October | -0.005 | 0.019 | 0.99 (0.96 – 1.03) | 0.797 | 0.025 | 0.023 | 1.03 (0.98 – 1.07) | 0.267 |
|  | November | 0.026 | 0.020 | 1.03 (0.99 – 1.07) | 0.178 | 0.031 | 0.023 | 1.03 (0.98 – 1.08) | 0.176 |
|  | December | 0.027 | 0.020 | 1.03 (0.99 – 1.07) | 0.173 | 0.051 | 0.023 | 1.05 (1.00 – 1.10) | 0.026 |
|  |  |  |  |  |  |  |  |  |  |
| Age group | Adult (2 - 6 years) (Ref.) |  |  |  |  |  |  |  |  |
|  | Geriatric (12+) | 0.316 | 0.013 | **1.37 (1.34 – 1.41)** | **< 0.001** | 0.274 | 0.015 | **1.31 (1.27 – 1.35)** | **< 0.001** |
|  | Puppy/Juvenile (0–1) | -0.424 | 0.018 | **0.66 (0.63 – 0.68)** | **< 0.001** | -0.448 | 0.022 | **0.64 (0.61 – 0.67)** | **< 0.001** |
|  | Senior (7–11) | 0.212 | 0.013 | **1.24 (1.21 – 1.27)** | **< 0.001** | 0.151 | 0.016 | **1.16 (1.12 – 1.20)** | **< 0.001** |
|  |  |  |  |  |  |  |  |  |  |
| Median household income | High (≥ $100k) (Ref.) |  |  |  |  |  |  |  |  |
|  | Low (< $40k) | 0.104 | 0.223 | 1.11 (0.72 – 1.72) | 0.642 | 0.039 | 0.263 | 1.04 (0.62 – 1.74) | 0.881 |
|  | Lower-Middle ($40k < $70k) | -0.073 | 0.031 | **0.93 (0.88 – 0.99)** | **0.019** | -0.089 | 0.040 | **0.92 (0.85 – 0.99)** | **0.027** |
|  | Upper-Middle ($70k < $100k) | -0.031 | 0.023 | 0.97 (0.92 – 1.02) | 0.260 | -0.032 | 0.035 | 0.97 (0.91 – 1.04) | 0.356 |
|  |  |  |  |  |  |  |  |  |  |
| Rural-Urban Continuum Codes | 1 (Ref.) |  |  |  |  |  |  |  |  |
|  | 2 | -0.020 | 0.022 | 1.02 (0.98 – 1.06) | 0.366 | 0.000 | 0.028 | 1.00 (0.95 – 1.06) | 0.999 |
|  | 3 | -0.045 | 0.027 | 0.96 (0.91 – 1.01) | 0.094 | -0.085 | 0.034 | **0.92 (0.86 – 0.99)** | **0.013** |
|  | 4 | 0.033 | 0.035 | 1.03 (0.97 – 1.11) | 0.336 | -0.040 | 0.043 | 0.96 (0.88 – 1.05) | 0.355 |
|  | 5 | 0.049 | 0.062 | 1.05 (0.92 – 1.20) | 0.450 | 0.022 | 0.081 | 1.02 (0.87 – 1.20) | 0.783 |
|  | 6 | 0.000 | 0.041 | 1.00 (0.92 – 1.08) | 0.996 | -0.068 | 0.051 | 0.93 (0.84 – 1.04) | 0.189 |
|  | 7 | 0.002 | 0.057 | 1.00 (0.90 – 1.11) | 0.972 | -0.037 | 0.071 | 0.96 (0.83 – 1.11) | 0.607 |
|  | 8 | 0.046 | 0.067 | 1.05 (0.92 – 1.11) | 0.493 | -0.107 | 0.084 | 0.90 (0.76 – 1.06) | 0.205 |
|  | 9 | -0.071 | 0.095 | 0.93 (0.77 – 1.02) | 0.455 | -0.251 | 0.123 | **0.78 (0.61 – 0.99)** | **0.041** |
|  |  |  |  |  |  |  |  |  |  |
| Sex | Female (Ref.) |  |  |  |  |  |  |  |  |
|  | Male | 0.021 | 0.026 | 1.02 (0.97 – 1.07) | 0.432 | 0.034 | 0.031 | 1.03 (0.97 – 1.10) | 0.272 |
|  |  |  |  |  |  |  |  |  |  |
| Number of housing units per veterinarian | 2000 - 3000 (Ref.) |  |  |  |  |  |  |  |  |
|  | 1000 - 1499 | -0.034 | 0.134 | 0.97 (0.74 – 1.26) | 0.797 | -0.104 | 0.179 | 0.90 (0.63 – 1.28) | 0.564 |
|  | 1500 - 1999 | 0.024 | 0.139 | 1.02 (0.78 – 1.34) | 0.861 | -0.033 | 0.186 | 0.97 (0.67 – 1.41) | 0.858 |
|  | 500 - 999 | -0.206 | 0.141 | 0.81 (0.62 – 1.07) | 0.144 | -0.320 | 0.189 | 0.73 (0.50 – 1.07) | 0.091 |
|  |  |  |  |  |  |  |  |  |  |
|  |  |  |  |  |  |  |  |  |  |
| Percentage tested |  | -0.010 | 0.001 | **0.99 (0.99 – 0.99)** | **< 0.001** | -0.010 | 0.001 | **0.99 (0.99 – 0.99)** | **< 0.001** |
|  |  |  |  |  |  |  |  |  |  |
| Number of prior positive cultures in the last 6 months | Isolates from patients without prior positive culture in the last 6 months (Ref.) |  |  |  |  |  |  |  |  |
|  | 1 | 0.253 | 0.014 | **1.29 (1.26 – 1.33)** | **< 0.001** | 0.242 | 0.016 | **1.27 (1.23 – 1.31)** | **< 0.001** |
|  | 2 | 0.538 | 0.030 | **1.71 (1.61 – 1.82)** | **< 0.001** | 0.531 | 0.033 | **1.70 (1.60 – 1.81)** | **< 0.001** |
|  | 3 | 0.862 | 0.065 | **2.37 (2.09– 2.69)** | **< 0.001** | 0.815 | 0.067 | **2.26 (1.98 – 2.57)** | **< 0.001** |
|  | 4 | 1.079 | 0.141 | **2.94 (2.22 – 3.89)** | **< 0.001** | 0.992 | 0.139 | **2.70 (2.05 – 3.56)** | **< 0.001** |
|  | 5 | 1.854 | 0.369 | **6.38 (3.11 – 13.08)** | **< 0.001** | 1.027 | 0.314 | **2.79 (1.51 – 5.15)** | **0.001** |
|  |  |  |  |  |  |  |  |  |  |
| Age group & sex interaction | Adult (2 - 6 years) & Female (Ref.) |  |  |  |  |  |  |  |  |
|  | Geriatric (12+) x Male | -0.115 | 0.030 | **0.89 (0.84 – 0.95)** | **< 0.001** | -0.184 | 0.035 | **0.83 (0.77 – 0.90)** | **< 0.001** |
|  | Puppy/Juvenile (0–1) x Male | 0.287 | 0.054 | **1.33 (1.19 – 1.49)** | **< 0.001** | 0.338 | 0.065 | **1.40 (1.23 – 1.59)** | **< 0.001** |
|  | Senior (7–11) x Male | -0.132 | 0.020 | **0.88 (0.83 – 0.93)** | **< 0.001** | -0.159 | 0.036 | **0.85 (0.79 – 0.92)** | **< 0.001** |

|  |  | **TRISUL** | | | | **CEFOV** | | | |
| --- | --- | --- | --- | --- | --- | --- | --- | --- | --- |
| Predictor | Category | Estimates | Std. Error | OR (95% CI) | p-value | Estimates | Std. Error | OR (95% CI) | p-value |
| Intercept |  | -2.476 | 0.265 | - | < 0.001 | -1.477 | 0.207 | - | < 0.001 |
| Year |  | -0.048 | 0.004 | **0.95 (0.95 – 0.96)** | **< 0.001** | -0.022 | 0.003 | **0.98 (0.97–0.98)** | **< 0.001** |
| Month | January (Ref.) |  |  |  |  |  |  |  |  |
|  | February | -0.007 | 0.036 | 0.99 (0.92 – 1.07) | 0.846 | -0.017 | 0.024 | 0.98 (0.93–1.04) | 0.496 |
|  | March | 0.008 | 0.035 | 1.01 (0.94 – 1.08) | 0.832 | -0.010 | 0.024 | 0.99 (0.93–1.05) | 0.696 |
|  | April | -0.008 | 0.037 | 0.99 (0.92 – 1.07) | 0.830 | -0.032 | 0.025 | 0.97 (0.92–1.03) | 0.215 |
|  | May | -0.032 | 0.036 | 0.97 (0.90 – 1.04) | 0.369 | -0.028 | 0.025 | 0.97 (0.92–1.03) | 0.265 |
|  | June | -0.028 | 0.035 | 0.97 (0.91 – 1.04) | 0.435 | -0.034 | 0.024 | 0.97 (0.92–1.03) | 0.157 |
|  | July | -0.052 | 0.035 | 0.95 (0.89 – 1.02) | 0.141 | -0.012 | 0.024 | 0.99 (0.94–1.04) | 0.609 |
|  | August | -0.020 | 0.034 | 0.98 (0.92 – 1.05) | 0.557 | 0.015 | 0.024 | 1.01 (0.96–1.06) | 0.537 |
|  | September | 0.007 | 0.035 | 1.01 (0.94 – 1.08) | 0.850 | 0.035 | 0.024 | 1.04 (0.98–1.10) | 0.149 |
|  | October | 0.017 | 0.035 | 1.02 (0.95 – 1.09) | 0.632 | 0.018 | 0.024 | 1.02 (0.96–1.07) | 0.443 |
|  | November | 0.025 | 0.035 | 1.03 (0.96 – 1.10) | 0.475 | 0.019 | 0.024 | 1.02 (0.97–1.08) | 0.435 |
|  | December | 0.017 | 0.035 | 1.02 (0.95 – 1.09) | 0.629 | 0.047 | 0.024 | 1.05 (0.99–1.10) | 0.055 |
|  |  |  |  |  |  |  |  |  |  |
| Age group | Adult (2 - 6 years) (Ref.) |  |  |  |  |  |  |  |  |
|  | Geriatric (12+) | 0.208 | 0.025 | **1.23 (1.17 – 1.29)** | **<0.001** | 0.319 | 0.016 | **1.38 (1.33–1.43)** | **< 0.001** |
|  | Puppy/Juvenile (0–1) | -0.122 | 0.031 | **0.89 (0.83 – 0.94)** | **<0.001** | -0.540 | 0.023 | 0.58 (0.55–0.61) | **< 0.001** |
|  | Senior (7–11) | 0.123 | 0.025 | **1.13 (1.08 – 1.19)** | **<0.001** | 0.181 | 0.017 | 1.20 (1.16–1.24) | **< 0.001** |
|  |  |  |  |  |  |  |  |  |  |
| Median household income | High (≥ $100k) (Ref.) |  |  |  |  |  |  |  |  |
|  | Low (< $40k) | -0.535 | 0.417 | 0.59 (0.26 – 1.33) | 0.200 | 0.092 | 0.269 | 1.10 (0.65–1.85) | 0.732 |
|  | Lower-Middle ($40k < $70k) | -0.102 | 0.054 | 0.90 (0.81 – 1.00) | 0.059 | -0.090 | 0.043 | **0.91 (0.84–0.99)** | **0.036** |
|  | Upper-Middle ($70k < $100k) | -0.060 | 0.046 | 0.94 (0.86 – 1.03) | 0.195 | -0.036 | 0.038 | 0.96 (0.89–1.04) | 0.339 |
|  |  |  |  |  |  |  |  |  |  |
| Rural-Urban Continuum Codes | 1 (Ref.) |  |  |  |  |  |  |  |  |
|  | 2 | -0.000 | 0.038 | 0.99 (0.93 – 1.08) | 0.985 | 0.006 | 0.030 | 1.01 (0.95–1.07) | 0.841 |
|  | 3 | 0.017 | 0.047 | 1.02 (0.93 – 1.12) | 0.711 | -0.064 | 0.037 | 0.94 (0.87–1.00) | 0.080 |
|  | 4 | 0.119 | 0.060 | **1.13 (1.00 – 1.27)** | **0.046** | 0.004 | 0.046 | 1.00 (0.92–1.09) | 0.937 |
|  | 5 | 0.108 | 0.115 | 1.11 (0.89 – 1.40) | 0.347 | -0.011 | 0.087 | 0.99 (0.84–1.18) | 0.904 |
|  | 6 | 0.149 | 0.071 | **1.16 (1.01 – 1.33)** | **0.037** | -0.040 | 0.055 | 0.96 (0.87–1.05) | 0.466 |
|  | 7 | 0.343 | 0.096 | **1.41 (1.17 – 1.70)** | **0.001** | 0.022 | 0.075 | 1.02 (0.88–1.18) | 0.771 |
|  | 8 | 0.031 | 0.123 | 1.03 (0.81 – 1.31) | 0.799 | -0.058 | 0.089 | 0.94 (0.79–1.11) | 0.515 |
|  | 9 | 0.070 | 0.178 | 1.07 (0.76 – 1.52) | 0.696 | -0.056 | 0.125 | 0.95 (0.74–1.20) | 0.656 |
|  |  |  |  |  |  |  |  |  |  |
| Sex | Female (Ref.) |  |  |  |  |  |  |  |  |
|  | Male | 0.162 | 0.047 | **1.18 (1.07 – 1.29)** | **<0.001** | -0.028 | 0.033 | 0.97 (0.91–1.03) | 0.409 |
|  |  |  |  |  |  |  |  |  |  |
| Number of housing units per veterinarian | 2000 - 3000 (Ref.) |  |  |  |  |  |  |  |  |
|  | 1000 - 1499 | -0.043 | 0.266 | 0.96 (0.57 – 1.61) | 0.872 | -0.157 | 0.209 | 0.86 (0.52–1.40) | 0.452 |
|  | 1500 - 1999 | 0.130 | 0.276 | 1.14 (0.66 – 1.96) | 0.638 | -0.084 | 0.217 | 0.92 (0.60–1.40) | 0.699 |
|  | 500 - 999 | -0.309 | 0.281 | 0.73 (0.42 – 1.27) | 0.272 | -0.412 | 0.220 | 0.66 (0.42–1.03) | 0.062 |
|  |  |  |  |  |  |  |  |  |  |
|  |  |  |  |  |  |  |  |  |  |
| Percentage tested |  | -0.015 | 0.002 | **0.99 (0.98 – 0.99)** | **< 0.001** | -0.010 | 0.001 | **0.99 (0.99–0.99)** | **< 0.001** |
|  |  |  |  |  |  |  |  |  |  |
| Number of prior positive cultures in the last 6 months | Isolates from patients without prior positive culture in the last 6 months (Ref.) |  |  |  |  |  |  |  |  |
|  | 1 | 0.539 | 0.022 | **1.71 (1.64 – 1.79)** | **< 0.001** | 0.288 | 0.016 | **1.33 (1.29–1.38)** | **< 0.001** |
|  | 2 | 0.928 | 0.042 | **2.53 (2.33 – 2.75)** | **< 0.001** | 0.628 | 0.034 | **1.87 (1.75–1.99)** | **< 0.001** |
|  | 3 | 1.166 | 0.083 | **3.21 (2.72 – 3.78)** | **< 0.001** | 0.959 | 0.068 | **2.61 (2.28–2.98)** | **< 0.001** |
|  | 4 | 1.389 | 0.164 | **4.01 (2.92 – 5.51)** | **< 0.001** | 1.026 | 0.140 | **2.79 (2.11–3.69)** | **< 0.001** |
|  | 5 | 1.235 | 0.370 | **3.44 (1.67 – 7.10)** | **< 0.001** | 1.092 | 0.322 | **2.98 (1.58–5.61)** | **< 0.001** |
|  |  |  |  |  |  |  |  |  |  |
| Age group & sex interaction | Adult (2 - 6 years) & Female (Ref.) |  |  |  |  |  |  |  |  |
|  | Geriatric (12+) x Male | -0.159 | 0.054 | **0.85 (0.77 – 0.95)** | **0.003** | -0.166 | 0.038 | **0.85 (0.79–0.92)** | **< 0.001** |
|  | Puppy/Juvenile (0–1) x Male | 0.266 | 0.092 | **1.31 (1.09 – 1.56)** | **0.002** | 0.417 | 0.071 | **1.52 (1.32–1.75)** | **< 0.001** |
|  | Senior (7–11) x Male | -0.124 | 0.055 | **0.88 (0.79 – 0.98)** | **0.022** | -0.145 | 0.039 | **0.86 (0.79–0.93)** | **< 0.001** |

|  |  | **CEFOD** | | | | **ENROF** | | | |
| --- | --- | --- | --- | --- | --- | --- | --- | --- | --- |
| Predictor | Category | Estimates | Std. Error | OR (95% CI) | p-value | Estimates | Std. Error | OR (95% CI) | p-value |
| Intercept |  | -1.634 | 0.217 | - | < 0.001 | -1.897 | 0.316 | - | < 0.001 |
| Year |  | -0.011 | 0.003 | **0.99 (0.98–0.99)** | **< 0.001** | -0.047 | 0.004 | **0.95 (0.94–0.95)** | **< 0.001** |
| Month | January (Ref.) |  |  |  |  |  |  |  |  |
|  | February | -0.011 | 0.026 | 1.00 (0.95–1.05) | 0.965 | -0.059 | 0.031 | 0.94 (0.89–1.00) | 0.056 |
|  | March | 0.012 | 0.025 | 1.01 (0.96–1.06) | 0.636 | -0.030 | 0.030 | 0.97 (0.92–1.03) | 0.319 |
|  | April | -0.003 | 0.026 | 1.00 (0.95–1.05) | 0.916 | -0.043 | 0.031 | 0.96 (0.90–1.02) | 0.169 |
|  | May | -0.001 | 0.026 | 1.00 (0.95–1.05) | 0.962 | -0.085 | 0.031 | **0.92 (0.86–0.98)** | **0.005** |
|  | June | 0.008 | 0.025 | 1.01 (0.96–1.06) | 0.738 | -0.109 | 0.030 | **0.90 (0.85–0.95)** | **< 0.001** |
|  | July | 0.011 | 0.025 | 1.01 (0.96–1.06) | 0.671 | -0.092 | 0.029 | **0.91 (0.86–0.97)** | **0.002** |
|  | August | 0.047 | 0.024 | 1.05 (1.00–1.10) | 0.056 | -0.098 | 0.030 | **0.91 (0.86–0.97)** | **< 0.001** |
|  | September | 0.058 | 0.025 | **1.06 (1.01–1.11)** | **0.019** | -0.013 | 0.030 | 0.99 (0.93–1.05) | 0.661 |
|  | October | 0.043 | 0.025 | 1.04 (0.99 – 1.10) | 0.083 | 0.016 | 0.029 | 1.02 (0.96–1.08) | 0.592 |
|  | November | 0.052 | 0.025 | **1.05 (1.00–1.11)** | **0.041** | 0.008 | 0.030 | 1.01 (0.95–1.07) | 0.781 |
|  | December | 0.070 | 0.025 | **1.07 (1.02–1.13)** | **0.005** | 0.000 | 0.030 | 1.00 (0.94–1.06) | 0.997 |
|  |  |  |  |  |  |  |  |  |  |
| Age group | Adult (2 - 6 years) (Ref.) |  |  |  |  |  |  |  |  |
|  | Geriatric (12+) | 0.280 | 0.017 | **1.32 (1.28–1.37)** | **< 0.001** | 0.474 | 0.021 | **1.61 (1.54–1.68)** | **< 0.001** |
|  | Puppy/Juvenile (0–1) | -0.487 | 0.024 | **0.62 (0.59–0.64)** | **< 0.001** | -0.769 | 0.034 | **0.46 (0.43–0.50)** | **< 0.001** |
|  | Senior (7–11) | 0.148 | 0.017 | **1.16 (1.12–1.20)** | **< 0.001** | 0.312 | 0.021 | **1.37 (1.32–1.43)** | **< 0.001** |
|  |  |  |  |  |  |  |  |  |  |
| Median household income | High (≥ $100k) (Ref.) |  |  |  |  |  |  |  |  |
|  | Low (< $40k) | 0.100 | 0.279 | 1.10 (0.64–1.90) | 0.733 | -0.586 | 0.389 | 0.56 (0.26–1.20) | 0.132 |
|  | Lower-Middle ($40k < $70k) | -0.110 | 0.050 | **0.90 (0.82–0.98)** | **0.016** | -0.101 | 0.064 | 0.90 (0.79–1.03) | 0.116 |
|  | Upper-Middle ($70k < $100k) | -0.037 | 0.040 | 0.96 (0.89–1.04) | 0.352 | -0.060 | 0.057 | 0.94 (0.84–1.05) | 0.293 |
|  |  |  |  |  |  |  |  |  |  |
| Rural-Urban Continuum Codes | 1 (Ref.) |  |  |  |  |  |  |  |  |
|  | 2 | -0.002 | 0.032 | 1.00 (0.94–1.06) | 0.954 | 0.044 | 0.045 | 1.05 (0.96–1.15) | 0.322 |
|  | 3 | -0.073 | 0.039 | 0.93 (0.86–1.00) | 0.058 | -0.017 | 0.053 | 0.98 (0.88–1.08) | 0.753 |
|  | 4 | -0.019 | 0.049 | 0.98 (0.89 – 1.08) | 0.699 | 0.036 | 0.066 | 1.04 (0.91–1.18) | 0.581 |
|  | 5 | -0.067 | 0.093 | 0.94 (0.78–1.12) | 0.471 | 0.192 | 0.118 | 1.21 (0.96–1.52) | 0.103 |
|  | 6 | -0.076 | 0.058 | 0.93 (0.83–1.04) | 0.189 | 0.0103 | 0.074 | 1.10 (0.95–1.28) | 0.190 |
|  | 7 | -0.029 | 0.080 | 0.97 (0.83–1.14) | 0.713 | 0.063 | 0.103 | 1.06 (0.87–1.30) | 0.542 |
|  | 8 | -0.084 | 0.094 | 0.92 (0.77–1.11) | 0.372 | 0.064 | 0.118 | 1.07 (0.85–1.35) | 0.590 |
|  | 9 | -0.159 | 0.134 | 0.85 (0.66–1.11) | 0.236 | 0.064 | 0.166 | 1.07 (0.77-1.48) | 0.699 |
|  |  |  |  |  |  |  |  |  |  |
| Sex | Female (Ref.) |  |  |  |  |  |  |  |  |
|  | Male | -0.001 | 0.034 | 1.00 (0.94–1.07) | 0.985 | 0.118 | 0.042 | **1.13 (1.04–1.23)** | **0.006** |
|  |  |  |  |  |  |  |  |  |  |
| Number of housing units per veterinarian | 2000 - 3000 (Ref.) |  |  |  |  |  |  |  |  |
|  | 1000 - 1499 | -0.135 | 0.219 | 0.87 (0.57–1.34) | 0.536 | -0.269 | 0.319 | 0.76 (0.41–1.39) | 0.399 |
|  | 1500 - 1999 | -0.069 | 0.227 | 0.93 (0.60–1.46) | 0.762 | -0.072 | 0.331 | 0.93(0.49–1.75) | 0.828 |
|  | 500 - 999 | -0.421 | 0.231 | 0.66 (0.42–1.03) | 0.068 | -0.596 | 0.337 | 0.55 (0.28–1.07) | 0.077 |
|  |  |  |  |  |  |  |  |  |  |
|  |  |  |  |  |  |  |  |  |  |
| Percentage tested |  | -0.009 | 0.002 | **0.99 (0.99–0.99)** | **< 0.001** | -0.016 | 0.002 | **0.98 (0.98–0.99)** | **< 0.001** |
|  |  |  |  |  |  |  |  |  |  |
| Number of prior positive cultures in the last 6 months | Isolates from patients without prior positive culture in the last 6 months (Ref.) |  |  |  |  |  |  |  |  |
|  | 1 | 0.242 | 0.017 | **1.27 (1.23–1.32)** | **< 0.001** | 0.725 | 0.019 | **2.07 (2.00–2.15)** | **< 0.001** |
|  | 2 | 0.554 | 0.035 | **1.74 (1.62–1.86)** | **< 0.001** | 1.247 | 0.037 | **3.48 (3.23–3.75)** | **< 0.001** |
|  | 3 | 0.865 | 0.070 | **2.37 (2.07–2.73)** | **< 0.001** | 1.626 | 0.077 | **5.08 (4.36–5.92)** | **< 0.001** |
|  | 4 | 0.853 | 0.144 | **2.35 (1.77–3.11)** | **< 0.001** | 1.986 | 0.160 | **7.28 (5.32–9.97)** | **< 0.001** |
|  | 5 | 0.962 | 0.328 | **2.62 (1.38–4.97)** | **0.003** | 2.122 | 0.377 | **8.35 (3.98–17.50)** | **< 0.001** |
|  |  |  |  |  |  |  |  |  |  |
| Age group & sex interaction | Adult (2 - 6 years) & Female (Ref.) |  |  |  |  |  |  |  |  |
|  | Geriatric (12+) x Male | -0.193 | 0.039 | **0.83 (0.77–0.89)** | **< 0.001** | -0.199 | 0.048 | **0.82 (0.75–0.89)** | **< 0.001** |
|  | Puppy/Juvenile (0–1) x Male | 0.407 | 0.071 | **1.50 (1.31–1.73)** | **< 0.001** | 0.303 | 0.097 | **1.35 (1.12–1.63)** | **< 0.001** |
|  | Senior (7–11) x Male | -0.163 | 0.039 | **0.85 (0.79–0.92)** | **< 0.001** | -0.197 | 0.049 | **0.82 (0.75–0.90)** | **< 0.001** |

|  |  | **MARBOF** | | | |
| --- | --- | --- | --- | --- | --- |
| Predictor | Category | Estimates | Std. Error | OR (95% CI) | p-value |
| Intercept |  | -2.110 | 0.382 | - | < 0.001 |
| Year |  | -0.076 | 0.004 | **0.93 (0.92–0.94)** | **< 0.001** |
| Month | January (Ref.) |  |  |  |  |
|  | February | -0.102 | 0.036 | **0.90 (0.84–0.96)** | **0.004** |
|  | March | -0.047 | 0.035 | 0.95 (0.89–1.02) | 0.174 |
|  | April | -0.073 | 0.036 | **0.93 (0.87–0.99)** | **0.042** |
|  | May | -0.113 | 0.035 | **0.89 (0.83–0.95)** | **0.001** |
|  | June | -0.117 | 0.035 | **0.89 (0.83–0.95)** | **< 0.001** |
|  | July | -0.093 | 0.034 | **0.91 (0.85–0.97)** | **0.007** |
|  | August | -0.106 | 0.034 | **0.90 (0.85–0.96)** | **0.002** |
|  | September | 0.003 | 0.034 | 1.00 (0.94–1.07) | 0.932 |
|  | October | -0.001 | 0.034 | 1.00 (0.94–1.06) | 0.974 |
|  | November | 0.009 | 0.034 | 1.01 (0.95–1.08) | 0.785 |
|  | December | -0.041 | 0.035 | 0.96 (0.90–1.03) | 0.230 |
|  |  |  |  |  |  |
| Age group | Adult (2 - 6 years) (Ref.) |  |  |  |  |
|  | Geriatric (12+) | 0.589 | 0.025 | **1.80 (1.71–1.89)** | **< 0.001** |
|  | Puppy/Juvenile (0–1) | -1.260 | 0.047 | **0.28 (0.26–0.30)** | **< 0.001** |
|  | Senior (7–11) | 0.394 | 0.025 | **1.48 (1.41–1.55)** | **< 0.001** |
|  |  |  |  |  |  |
| Median household income | High (≥ $100k) (Ref.) |  |  |  |  |
|  | Low (< $40k) | -0.599 | 0.443 | 0.55 (0.23–1.33) | 0.176 |
|  | Lower-Middle ($40k < $70k) | -0.078 | 0.077 | 0.92 (0.79–1.07) | 0.307 |
|  | Upper-Middle ($70k < $100k) | -0.050 | 0.068 | 0.95 (0.83–1.09) | 0.465 |
|  |  |  |  |  |  |
| Rural-Urban Continuum Codes | 1 (Ref.) |  |  |  |  |
|  | 2 | 0.044 | 0.053 | 1.05 (0.95–1.16) | 0.409 |
|  | 3 | -0.026 | 0.063 | 0.97 (0.86–1.10) | 0.675 |
|  | 4 | 0.061 | 0.077 | 1.06 (0.91–1.23) | 0.434 |
|  | 5 | 0.198 | 0.140 | 1.22 (0.93–1.61) | 0.156 |
|  | 6 | 0.090 | 0.087 | 1.09 (0.92–1.29) | 0.303 |
|  | 7 | 0.164 | 0.119 | 1.18 (0.94–1.48) | 0.168 |
|  | 8 | -0.051 | 0.143 | 0.95 (0.72–1.25) | 0.722 |
|  | 9 | 0.158 | 0.191 | 1.17 (0.81–1.69) | 0.409 |
|  |  |  |  |  |  |
| Sex | Female (Ref.) |  |  |  |  |
|  | Male | 0.187 | 0.049 | **1.21 (1.10–1.33)** | **< 0.001** |
|  |  |  |  |  |  |
| Number of housing units per veterinarian | 2000 - 3000 (Ref.) |  |  |  |  |
|  | 1000 - 1499 | -0.361 | 0.387 | 0.70 (0.33–1.50) | 0.351 |
|  | 1500 - 1999 | -0.160 | 0.402 | 0.85 (0.38–1.92) | 0.691 |
|  | 500 - 999 | -0.774 | 0.409 | **0.46 (0.20–1.06)** | **0.058** |
|  |  |  |  |  |  |
|  |  |  |  |  |  |
| Percentage tested |  | -0.020 | 0.002 | **0.98 (0.97–0.98)** | **< 0.001** |
|  |  |  |  |  |  |
| Number of prior positive cultures in the last 6 months | Isolates from patients without prior positive culture in the last 6 months (Ref.) |  |  |  |  |
|  | 1 | 0.761 | 0.021 | **2.14 (2.05–2.23)** | **< 0.001** |
|  | 2 | 1.278 | 0.041 | **3.59 (3.32–3.88)** | **< 0.001** |
|  | 3 | 1.726 | 0.082 | **5.62 (4.80–6.58)** | **< 0.001** |
|  | 4 | 1.891 | 0.167 | **6.63 (4.78–9.19)** | **< 0.001** |
|  | 5 | 2.525 | 0.387 | **12.50 (5.83 – 26.82)** | **< 0.001** |
|  |  |  |  |  |  |
| Age group & sex interaction | Adult (2 - 6 years) & Female (Ref.) |  |  |  |  |
|  | Geriatric (12+) x Male | -0.259 | 0.056 | **0.77 (0.69–0.87)** | **< 0.001** |
|  | Puppy/Juvenile (0–1) x Male | 0.656 | 0.121 | **1.93 (1.52–2.44)** | **< 0.001** |
|  | Senior (7–11) x Male | -0.259 | 0.056 | **0.77 (0.69-0.86)** | **< 0.001** |

Model included year, month, age, sex, age–sex interaction, Rural-Urban Continuum Codes, number of housing units per veterinarian, Percentage tested, number of prior positive cultures in the last 6 months, and median household income, with random effects at the state, county, and patient levels.

AMOXI-Amoxicillin, AMOCLA-Amoxicillin clavulanic acid, TRISUL-Trimethoprim-sulfamethoxazole, CEFOV-Cefovecin, CEFPOD-Cefpodoxime, ENROF-Enrofloxacin, MARBOF-Marbofloxacin. Inverse OR values (1/OR) aid interpretation when the reference category is reversed.

**Supplementary Table 8.** Direct effect of Rural-Urban Continuum Codes on antimicrobial resistance in urinary *E. coli* isolates from dogs in the USA (2018–2024)

| **Antimicrobial** | **Rural-Urban Continuum Codes** | **Estimates** | **Standard Error** | **OR (95% CI)** | **p-value** | **Partial χ²** | **df** | **χ²/df** | **Partial likelihood ratio test** |
| --- | --- | --- | --- | --- | --- | --- | --- | --- | --- |
|  |  |  |  |  |  |  |  |  | 0.199 |
|  | 1 | Ref. |  |  |  |  |  |  |  |
|  | 2 | -0.020 | 0.022 | 1.02 (0.98 – 1.06) | 0.366 |  |  |  |  |
|  | 3 | -0.045 | 0.027 | 0.96 (0.91 – 1.01) | 0.094 |  |  |  |  |
| AMOXI | 4 | 0.033 | 0.035 | 1.03 (0.97 – 1.11) | 0.336 | 11.04 | 8 | 1.38 |  |
|  | 5 | 0.049 | 0.062 | 1.05 (0.92 – 1.20) | 0.450 |  |  |  |  |
|  | 6 | 0.000 | 0.041 | 1.00 (0.92 – 1.08) | 0.996 |  |  |  |  |
|  | 7 | 0.002 | 0.057 | 1.00 (0.90 – 1.11) | 0.972 |  |  |  |  |
|  | 8 | 0.046 | 0.067 | 1.05 (0.92 – 1.11) | 0.493 |  |  |  |  |
|  | 9 | -0.071 | 0.095 | 0.93 (0.77 – 1.02) | 0.455 |  |  |  |  |
|  |  |  |  |  |  |  |  |  | **0.030** |
|  | 1 | Ref. |  |  |  |  |  |  |  |
|  | 2 | 0.000 | 0.028 | 1.00 (0.95 – 1.06) | 0.999 |  |  |  |  |
|  | 3 | -0.085 | 0.034 | **0.92 (0.86 – 0.99)** | **0.013** |  |  |  |  |
| AMOCLA | 4 | -0.040 | 0.043 | 0.96 (0.88 – 1.05) | 0.355 | 16.97 | 8 | 2.12 |  |
|  | 5 | 0.022 | 0.081 | 1.02 (0.87 – 1.20) | 0.783 |  |  |  |  |
|  | 6 | -0.068 | 0.051 | 0.93 (0.84 – 1.04) | 0.189 |  |  |  |  |
|  | 7 | -0.037 | 0.071 | 0.96 (0.83 – 1.11) | 0.607 |  |  |  |  |
|  | 8 | -0.107 | 0.084 | 0.90 (0.76 – 1.06) | 0.205 |  |  |  |  |
|  | 9 | -0.251 | 0.123 | **0.78 (0.61 – 0.99)** | **0.041** |  |  |  |  |
|  |  |  |  |  |  |  |  |  | **0.005** |
|  | 1 | Ref. |  |  |  |  |  |  |  |
|  | 2 | -0.000 | 0.038 | 0.99 (0.93 – 1.08) | 0.985 |  |  |  |  |
|  | 3 | 0.017 | 0.047 | 1.02 (0.93 – 1.12) | 0.711 |  |  |  |  |
| TRISUL | 4 | 0.119 | 0.060 | 1.13 (1.00 – 1.27) | 0.046 | 22.00 | 8 | 2.75 |  |
|  | 5 | 0.108 | 0.115 | 1.11 (0.89 – 1.40) | 0.347 |  |  |  |  |
|  | 6 | 0.149 | 0.071 | **1.16 (1.01 – 1.33)** | **0.037** |  |  |  |  |
|  | 7 | 0.343 | 0.096 | **1.41 (1.17 – 1.70)** | **0.001** |  |  |  |  |
|  | 8 | 0.031 | 0.123 | 1.03 (0.81 – 1.31) | 0.799 |  |  |  |  |
|  | 9 | 0.070 | 0.178 | 1.07 (0.76 – 1.52) | 0.696 |  |  |  |  |
|  |  |  |  |  |  |  |  |  | 0.532 |
|  | 1 | Ref. |  |  |  |  |  |  |  |
|  | 2 | 0.006 | 0.030 | 1.01 (0.95–1.07) | 0.841 |  |  |  |  |
|  | 3 | -0.064 | 0.037 | 0.94 (0.87–1.00) | 0.080 |  |  |  |  |
| CEFOV | 4 | 0.004 | 0.046 | 1.00 (0.92–1.09) | 0.937 |  |  |  |  |
|  | 5 | -0.011 | 0.087 | 0.99 (0.84–1.18) | 0.904 | 7.04 | 8 | 0.88 |  |
|  | 6 | -0.040 | 0.055 | 0.96 (0.87–1.05) | 0.466 |  |  |  |  |
|  | 7 | 0.022 | 0.075 | 1.02 (0.88–1.18) | 0.771 |  |  |  |  |
|  | 8 | -0.058 | 0.089 | 0.94 (0.79–1.11) | 0.515 |  |  |  |  |
|  | 9 | -0.056 | 0.125 | 0.95 (0.74–1.20) | 0.656 |  |  |  |  |
|  |  |  |  |  |  |  |  |  | 0.294 |
|  | 1 | Ref. |  |  |  |  |  |  |  |
|  | 2 | -0.002 | 0.032 | 1.00 (0.94–1.06) | 0.954 |  |  |  |  |
|  | 3 | -0.073 | 0.039 | 0.93 (0.86–1.00) | 0.058 |  |  |  |  |
| CEFPOD | 4 | -0.019 | 0.049 | 0.98 (0.89 – 1.08) | 0.699 |  |  |  |  |
|  | 5 | -0.067 | 0.093 | 0.94 (0.78–1.12) | 0.471 | 9.60 | 8 | 1.20 |  |
|  | 6 | -0.076 | 0.058 | 0.93 (0.83–1.04) | 0.189 |  |  |  |  |
|  | 7 | -0.029 | 0.080 | 0.97 (0.83–1.14) | 0.713 |  |  |  |  |
|  | 8 | -0.084 | 0.094 | 0.92 (0.77–1.11) | 0.372 |  |  |  |  |
|  | 9 | -0.159 | 0.134 | 0.85 (0.66–1.11) | 0.236 |  |  |  |  |
|  |  |  |  |  |  |  |  |  | 0.608 |
|  | 1 | Ref. |  |  |  |  |  |  |  |
|  | 2 | 0.044 | 0.045 | 1.05 (0.96–1.15) | 0.322 |  |  |  |  |
|  | 3 | -0.017 | 0.053 | 0.98 (0.88–1.08) | 0.753 | 6.35 | 8 | 0.79 |  |
| ENROF | 4 | 0.036 | 0.066 | 1.04 (0.91–1.18) | 0.581 |  |  |  |  |
|  | 5 | 0.192 | 0.118 | 1.21 (0.96–1.52) | 0.103 |  |  |  |  |
|  | 6 | 0.0103 | 0.074 | 1.10 (0.95–1.28) | 0.190 |  |  |  |  |
|  | 7 | 0.063 | 0.103 | 1.06 (0.87–1.30) | 0.542 |  |  |  |  |
|  | 8 | 0.064 | 0.118 | 1.07 (0.85–1.35) | 0.590 |  |  |  |  |
|  | 9 | 0.064 | 0.166 | 1.07 (0.77-1.48) | 0.699 |  |  |  |  |
|  |  |  |  |  |  |  |  |  | 0.455 |
|  | 1 | Ref. |  |  |  |  |  |  |  |
|  | 2 | 0.044 | 0.053 | 1.05 (0.95–1.16) | 0.409 |  |  |  |  |
|  | 3 | -0.026 | 0.063 | 0.97 (0.86–1.10) | 0.675 |  |  |  |  |
| MARBOF | 4 | 0.061 | 0.077 | 1.06 (0.91–1.23) | 0.434 | 7.78 | 8 | 0.97 |  |
|  | 5 | 0.198 | 0.140 | 1.22 (0.93–1.61) | 0.156 |  |  |  |  |
|  | 6 | 0.090 | 0.087 | 1.09 (0.92–1.29) | 0.303 |  |  |  |  |
|  | 7 | 0.164 | 0.119 | 1.18 (0.94–1.48) | 0.168 |  |  |  |  |
|  | 8 | -0.051 | 0.143 | 0.95 (0.72–1.25) | 0.722 |  |  |  |  |
|  | 9 | 0.158 | 0.191 | 1.17 (0.81–1.69) | 0.409 |  |  |  |  |

Model adjusted for year, month, age, sex, age and sex interaction, median household income, Percentage tested, number of housing units per veterinarian, and number of prior positive cultures within the preceding 6 months with random effects at the state, county, and patient levels.

χ² - Chi-square statistic

df - degrees of freedom

AMOXI-Amoxicillin, AMOCLA-Amoxicillin clavulanic acid, TRISUL-Trimethoprim-sulfamethoxazole, CEFOV-Cefovecin, CEFPOD-Cefpodoxime, ENROF-Enrofloxacin, MARBOF-Marbofloxacin. Inverse OR values (1/OR) aid interpretation when the reference category is reversed.

**Supplementary Table 9**. Direct effect of household median household income at the county-level on antimicrobial resistance in urinary *E. coli* isolates from dogs in the USA (2018–2024)

| **Antimicrobial** | **Median household income** | **Estimates** | **Standard**  **Error** | **OR (95% CI)** | **p-value** | **Partial χ²** | **df** | **χ²/df** | **Partial likelihood ratio test** |
| --- | --- | --- | --- | --- | --- | --- | --- | --- | --- |
|  |  |  |  |  |  |  |  |  | **0.030** |
|  | High (≥ $100k) | Ref. |  |  |  |  |  |  |  |
| AMOXI | Low (< $40k) | 0.104 | 0.223 | 1.11 (0.72 – 1.72) | 0.642 | 8.96 | 3 | 2.99 |  |
|  | Lower-Middle ($40k < $70k) | -0.073 | 0.031 | **0.93 (0.88 – 0.99)** | **0.019** |  |  |  |  |
|  | Upper-Middle ($70k < $100k) | -0.031 | 0.023 | 0.97 (0.92 – 1.02) | 0.260 |  |  |  |  |
|  |  |  |  |  |  |  |  |  | **0.037** |
|  | High (≥ $100k) | Ref. |  |  |  |  |  |  |  |
| AMOCLA | Low (< $40k) | 0.039 | 0.263 | 1.04 (0.62 – 1.74) | 0.881 |  |  |  |  |
|  | Lower-Middle ($40k < $70k) | -0.089 | 0.040 | **0.92 (0.85 – 0.99)** | **0.027** | 8.51 | 3 | 2.84 |  |
|  | Upper-Middle ($70k < $100k) | -0.032 | 0.035 | 0.97 (0.91 – 1.04) | 0.356 |  |  |  |  |
|  |  |  |  |  |  |  |  |  | 0.104 |
|  | High (≥ $100k) | Ref. |  |  |  |  |  |  |  |
| TRISUL | Low (< $40k) | -0.535 | 0.417 | 0.59 (0.26 – 1.33) | 0.200 | 6.16 | 3 | 2.05 |  |
|  | Lower-Middle ($40k < $70k) | -0.102 | 0.054 | 0.90 (0.81 – 1.00) | 0.059 |  |  |  |  |
|  | Upper-Middle ($70k < $100k) | -0.060 | 0.046 | 0.94 (0.86 – 1.03) | 0.195 |  |  |  |  |
|  |  |  |  |  |  |  |  |  | 0.057 |
|  | High (≥ $100k) | Ref. |  |  |  |  |  |  |  |
| CEFOV | Low (< $40k) | 0.092 | 0.269 | 1.10 (0.65–1.85) | 0.732 | 7.04 | 3 | 2.50 |  |
|  | Lower-Middle ($40k < $70k) | -0.090 | 0.043 | 0.91 (0.84–0.99) | 0.036 |  |  |  |  |
|  | Upper-Middle ($70k < $100k) | -0.036 | 0.038 | 0.96 (0.89–1.04) | 0.339 |  |  |  |  |
|  |  |  |  |  |  |  |  |  | **0.012** |
|  | High (≥ $100k) | Ref. |  |  |  | 10.98 | 3 | 3.66 |  |
|  | Low (< $40k) | 0.100 | 0.279 | 1.10 (0.64–1.90) | 0.733 |  |  |  |  |
| CEFPOD | Lower-Middle ($40k < $70k) | -0.110 | 0.050 | **0.90 (0.82–0.98)** | **0.016** |  |  |  |  |
|  | Upper-Middle ($70k < $100k) | -0.037 | 0.040 | 0.96 (0.89–1.04) | 0.352 |  |  |  |  |
|  |  |  |  |  |  |  |  |  | 0.110 |
|  | High (≥ $100k) | Ref. |  |  |  |  |  |  |  |
| ENROF | Low (< $40k) | -0.586 | 0.389 | 0.56 (0.26–1.20) | 0.132 | 6.03 | 3 | 2.01 |  |
|  | Lower-Middle ($40k < $70k) | -0.101 | 0.064 | 0.90 (0.79–1.03) | 0.116 |  |  |  |  |
|  | Upper-Middle ($70k < $100k) | -0.060 | 0.057 | 0.94 (0.84–1.05) | 0.293 |  |  |  |  |
|  |  |  |  |  |  |  |  |  | 0.276 |
|  | High (≥ $100k) | Ref. |  |  |  |  |  |  |  |
| MARBOF | Low (< $40k) | -0.599 | 0.443 | 0.55 (0.23–1.33) | 0.176 | 3.87 | 3 | 1.29 |  |
|  | Lower-Middle ($40k < $70k) | -0.078 | 0.077 | 0.92 (0.79–1.07) | 0.307 |  |  |  |  |
|  | Upper-Middle ($70k < $100k) | -0.050 | 0.068 | 0.95 (0.83–1.09) | 0.465 |  |  |  |  |

Model adjusted for year, month, age, sex, age and sex interaction, Rural-Urban Continuum Codes, Percentage tested, Number of housing units per veterinarian, and number of prior positive cultures within the preceding 6 months with random effects at the state, county, and patient levels.

χ² - Chi-square statistic

df - degrees of freedom

AMOXI-Amoxicillin, AMOCLA-Amoxicillin clavulanic acid, TRISUL-Trimethoprim-sulfamethoxazole, CEFOV-Cefovecin, CEFPOD-Cefpodoxime, ENROF-Enrofloxacin, MARBOF-Marbofloxacin. Inverse OR values (1/OR) aid interpretation when the reference category is reversed.

**Supplementary Table 10**. Pairwise comparison of the number of housing units per veterinarian at the state-level on antimicrobial resistance in urinary *E. coli* isolates from dogs in the USA (2018–2024)

|  |  | **AMOXI** | | | **AMOCLA** | | | **TRISUL** | | |
| --- | --- | --- | --- | --- | --- | --- | --- | --- | --- | --- |
| Contrast | | OR | 95% CI (OR) | Adjusted p-value | OR | 95% CI (OR) | Adjusted p-value | OR | 95% CI (OR) | Adjusted p-value |
| 2,000–3,000 vs 1,000–1,499 | | 1.04 | 0.80 – 1.35 | 0.994 | 1.11 | 0.78 – 1.57 | 0.939 | 1.04 | 0.62 – 1.74 | 0.999 |
| 2,000–3,000 vs 1,500–1,999 | | 0.98 | 0.75 – 1.29 | 0.998 | 1.03 | 0.72 – 1.48 | 0.998 | 0.88 | 0.51 – 1.51 | 0.966 |
| 2,000–3,000 vs 500–999 | | 1.23 | 0.93 – 1.62 | 0.461 | 1.38 | 0.95 – 2.00 | 0.328 | 1.36 | 0.79 – 2.35 | 0.691 |
| 1,000–1,499 vs 1,500–1,999 | | 0.94 | 0.84 – 1.05 | 0.739 | 0.93 | 0.80 – 1.09 | 0.809 | 0.84 | 0.67 – 1.06 | 0.455 |
| 1,000–1,499 vs 500–999 | | **1.19** | **1.05 – 1.35** | **0.034** | 1.24 | 1.01 – 1.51 | 0.058 | 1.30 | 1.01 – 1.68 | 0.170 |
| 1,500–1,999 vs 500–999 | | **1.26** | **1.09 – 1.45** | **0.009** | **1.33** | **1.09 – 1.61** | **0.020** | **1.55** | **1.15 – 2.08** | **0.017** |
|  |  |  |  |  |  |  |  |  |  |  |

|  |  | **CEFOV** | | | **CEFPOD** | | | **ENROF** | | |
| --- | --- | --- | --- | --- | --- | --- | --- | --- | --- | --- |
| Contrast | | OR | 95% CI (OR) | Adjusted p-value | OR | 95% CI (OR) | Adjusted p-value | OR | 95% CI (OR) | Adjusted p-value |
| 2,000–3,000 vs 1,000–1,499 | | 1.17 | 0.78 – 1.75 | 0.876 | 1.14 | 0.74 – 1.76 | 0.926 | 1.31 | 0.70 – 2.45 | 0.834 |
| 2,000–3,000 vs 1,500–1,999 | | 1.09 | 0.71 – 1.67 | 0.980 | 1.07 | 0.69 – 1.67 | 0.990 | 1.07 | 0.56 – 2.06 | 0.996 |
| 2,000–3,000 vs 500–999 | | 1.51 | 0.98 – 2.34 | 0.242 | 1.52 | 0.95 – 2.42 | 0.262 | 1.82 | 0.94 – 3.51 | 0.289 |
| 1,000–1,499 vs 1,500–1,999 | | 0.93 | 0.78 – 1.11 | 0.860 | 0.94 | 0.78 – 1.13 | 0.903 | 0.82 | 0.62 – 1.09 | 0.515 |
| 1,000–1,499 vs 500–999 | | 1.29 | 1.00 – 1.66 | 0.060 | **1.33** | **1.02 – 1.73** | **0.037** | 1.39 | 1.02 – 1.89 | 0.157 |
| 1,500–1,999 vs 500–999 | | **1.39** | **1.10 – 1.75** | **0.026** | **1.42** | **1.11 – 1.81** | **0.021** | **1.69** | **1.19 – 2.40** | **0.019** |

|  |  | **MARBOF** | | |
| --- | --- | --- | --- | --- |
| Contrast | | OR | 95% CI (OR) | Adjusted p-value |
| 2,000–3,000 vs 1,000–1,499 | | 1.44 | 0.67 – 3.09 | 0.787 |
| 2,000–3,000 vs 1,500–1,999 | | 1.17 | 0.53 – 2.58 | 0.979 |
| 2,000–3,000 vs 500–999 | | 2.17 | 0.98 – 4.81 | 0.231 |
| 1,000–1,499 vs 1,500–1,999 | | 0.82 | 0.58 – 1.16 | 0.658 |
| 1,000–1,499 vs 500–999 | | 1.51 | 1.04 – 2.18 | 0.133 |
| 1,500–1,999 vs 500–999 | | **1.85** | **1.20 – 2.85** | **0.027** |

Model adjusted for year, month, age, sex, age and sex interaction, Median household income, Rural-Urban Continuum Codes, Percentage tested, and number of prior positive cultures within the preceding 6 months, with random effects at the state, county, and patient levels

AMOXI-Amoxicillin, AMOCLA-Amoxicillin clavulanic acid, TRISUL-Trimethoprim-sulfamethoxazole, CEFOV-Cefovecin, CEFPOD-Cefpodoxime, ENROF-Enrofloxacin, MARBOF-Marbofloxacin. Inverse OR values (1/OR) aid interpretation when the reference category is reversed.
